# A neuroprosthesis for restoring hand movement and sensation in a person with complete tetraplegia

**DOI:** 10.1038/s41591-026-04498-0

**Published:** 2026-07-16

**Authors:** Santosh Chandrasekaran, Sarah K. Wandelt, Aniket Jangam, Zeev Elias, Erona Ibroci, Christina Maffei, Isabelle A. Rosenthal, Richard Ramdeo, Joo-won Kim, Junqian Xu, Matthew F. Glasser, Allison Neuwirth, Todd A. Goldstein, Nathan E. Crone, Matthew S. Fifer, Gelana Tostaeva, Stephan Bickel, Douglas Griffin, Michael Funaro, Nicholas G. Carras, Rachel Pruitt, Netanel Ben-Shalom, Adam B. Stein, Ashesh D. Mehta, Chad E. Bouton

**Affiliations:** 1 Feinstein Institutes for Medical Research, Northwell Health, Manhasset, NY USA; 2 Northwell Health STARS Rehabilitation, Northwell Health, East Meadow, NY USA; 3 Department of Radiology and Psychiatry, Baylor College of Medicine, Houston, TX USA; 4 Department of Neuroscience, Washington University Medical School, Saint Louis, MO USA; 5 Northwell Center for Learning and Innovation, Northwell Health, Manhasset, NY USA; 6 Department of Neurology, Johns Hopkins Medical Institutions, Baltimore, MD USA; 7 Research and Exploratory Development Department, Johns Hopkins Applied Physics Laboratory, Laurel, MD USA; 8 North Shore University Department of Neurosurgery, Northwell Health, Manhasset, NY USA; 9 Donald and Barbara Zucker School of Medicine at Hofstra/Northwell, Manhasset, NY USA; 10 Lenox Hill Hospital Department of Neurosurgery, New York, NY USA; 11 Department of Physical Medicine and Rehabilitation, Northwell Health, Manhasset, NY USA

**Keywords:** Neurological disorders, Movement disorders

## Abstract

Millions of people worldwide are living with movement and sensory impairments owing to spinal cord injury, stroke and other neurological conditions. Here we report a double neural bypass (DNB), a hybrid neuroprosthetic system designed to restore both immediate and lasting gains in movement and sensation after a severe, complete spinal cord injury. The DNB links an intracortical brain–computer interface with targeted and patterned neuromodulation of the spinal cord and cortex. This allows brain signals associated with movement intention to directly control the movement of the user’s own hand in real time while also promoting long-term sensorimotor recovery—even after the system is turned off. The DNB system uses recurrent artificial neural networks and reinforcement learning for fine grasp control, together with patterned spinal cord stimulation and activity-informed intracortical microstimulation (‘cortical mirroring’) to promote neuroplasticity and durable recovery of function. In a participant with chronic C4 sensory/C5 motor complete tetraplegia, this hybrid approach enabled recovery of functional abilities including self-feeding and manipulation of delicate objects, while also producing significant and persistent improvements in elbow flexion and wrist tactile sensation. These findings demonstrate the potential of combining a sensorimotor neuroprosthesis with targeted brain and spinal neuromodulation to restore clinically relevant function in severe paralysis.

## Abstract (web summary)

A bidirectional sensorimotor neuroprosthetic system that decodes brain signals associated with movement intention while delivering patterned neuromodulation to the spinal cord and cortex provides long-term recovery of hand motor and sensory function, even when the device is off.

## Main

Spinal cord injury (SCI) is a leading cause of paralysis, with tetraplegia occurring in over half of all cases. Individuals with SCI consistently rank regaining upper limb movement as their highest recovery priority^1,2^. Furthermore, complete injuries, where no voluntary motor or sensory function is present below the level of injury, are particularly difficult to treat and recovery of useful function is exceedingly rare^3^. We, and subsequently other researchers, demonstrated that a motor neuroprosthesis using a unidirectional intracortical brain–computer interface (iBCI) linked to real-time muscle stimulation can restore thought-driven functional hand movement in complete tetraplegia^4,5^. However, this approach did not include tactile sensory feedback, important for skilled grasping, or methods to promote neuroplasticity and persistent sensorimotor recovery. Tactile feedback via intracortical microstimulation (ICMS) of the primary somatosensory cortex (S1)^6–10^ has been previously used to perform tasks with robotic manipulators^11,12^ but not with the user’s own hand. Furthermore, while temporary enhancement of tactile sensation using ICMS has been demonstrated, persistent recovery has remained elusive^13^. Lastly, iBCI-mediated spinal cord stimulation (SCS) has been demonstrated to recover function, albeit restricted to the lower limbs in incomplete SCI^14^.

**Extended Data Fig. 1:**
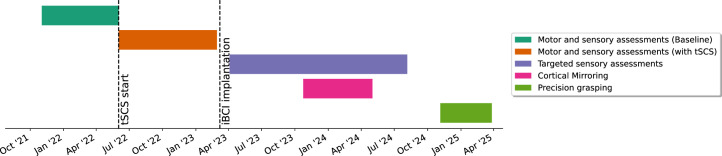
Study timeline. This shows the different phases of the study presented in this work and the timelines of data acquisition for each of the study experiments. The participant sustained a SCI 13 months prior to the start of the study.

To address the unmet clinical need of restoring upper limb function in individuals with high-level, complete SCIs, we developed a ‘double neural bypass’ (DNB)—a hybrid brain–body interface that provides both assistive and therapeutic benefits. To achieve this, the DNB integrates a bidirectional iBCI that records from, and stimulates in, the sensorimotor cortex, a stable neural decoding architecture to infer user intentions, deep reinforcement learning (RL) to support precision movements and transcutaneous SCS (tSCS) to modulate targeted dorsal spinal roots for promoting neuroplasticity and persistent recovery. In our first-in-human DNB clinical trial spanning more than 3 years (Extended Data Fig. 1), we enrolled a 42-year-old man with complete C4 sensory/C5 motor tetraplegia resulting from a diving accident 13 months prior. With an inability to lift his arms or hands to his face, hold objects or feel any sensation in his distal forearms and hands, his upper limb sensorimotor functions were severely limited.

## Overview of the DNB

**Fig. 1:**
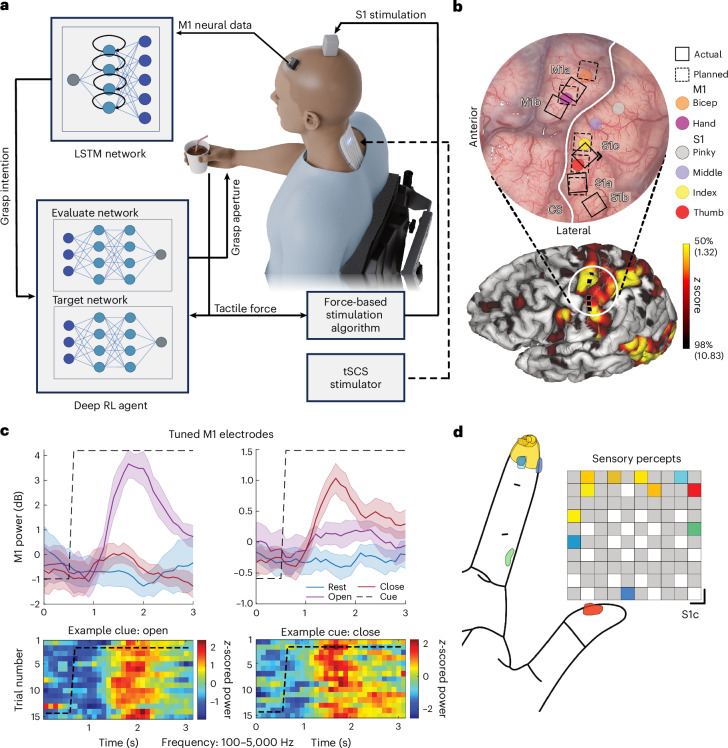
The DNB system. **a**, Schematic showing the DNB system. **b**, Top: intraoperative image of the brain showing M1 and S1 with the central sulcus (CS; white). Colored circles in M1 show intraoperative cortical stimulation sites that evoked EMG activation in the right BB (orange) and twitches in the right hand (magenta). Colored circles in S1 show awake intraoperative cortical stimulation sites that evoked sensory percepts in the thumb (red), index (yellow), middle (purple) and pinky (gray) fingers. Dashed and solid squares show planned and actual locations for array implantation, respectively. The black notch in the corner marks the medial sensory array (S1c). Bottom: task fMRI response (*z* score) to video-guided imagined and attempted thumb flexion and extension. Black squares are the planned array implantation sites shown on the pial surface using Connectome Workbench, overlaid on cortical areal boundaries from HCP cortical parcellation. **c**, Examples of M1 electrodes tuned to ‘open’ the hand (left) and ‘close’ the hand (right). Top: mean integrated broadband power (100–5,000 Hz) in M1 with shaded region showing 95% CI (*n* = 15 trials). Bottom: *z-*scored power depicting each trial. **d**, Example somatosensory percepts elicited by S1-ICMS shown to be localized to the thumb and index fingers. Each evoked percept is matched by color to the electrode that elicited it upon stimulation in the inset array. The black notch on the bottom right corner marks the medial sensory array (S1c) as in **b** (top). The array schematic also indicates electrodes that did not evoke a sensory percept (white squares) and electrodes that were not wired at all (gray squares). The other two sensory arrays (S1a and S1b) did not evoke any sensory percepts.

To restore upper limb functional ability in our study participant, the DNB utilizes several key subsystems that serve the assistive and/or therapeutic modalities. To restore cortically mediated high-precision hand grasping (assistive mode), the DNB uses multiple neural networks (Fig. 1a) in a nested closed-loop control architecture that utilizes a long short-term memory (LSTM) neural network and a deep-RL agent. Here, movement intentions are decoded from neural activity recorded via two microelectrode arrays implanted in the primary motor cortex (M1) while specific stimulation patterns are delivered to up to three microelectrode arrays implanted in S1 (Fig. 1b). Functional magnetic resonance imaging (fMRI) and cortical parcellation from the Human Connectome Project (HCP)^15^ was used to localize cortical regions of hand representation and plan implantation locations before surgery, with intraoperative cortical stimulation refining the target locations (Fig. 1b). Example M1 intracortical activity used as LSTM inputs for different hand movements are shown in Fig. 1c. Tactile sensory percepts evoked were localized mostly to the index and thumb pad and the corresponding electrodes that were stimulated are shown in Fig. 1d. For both motor and sensory therapeutic modalities, tSCS is delivered through a custom electrode patch^16^ (Fig. 1a). For sensory intervention, the iBCI leverages its bidirectional approach to record from, and stimulate in, S1 by attempting to activate similar cortical circuits that are active during imagined sensation.

## Initial SCS and sensorimotor baselining

**Fig. 2:**
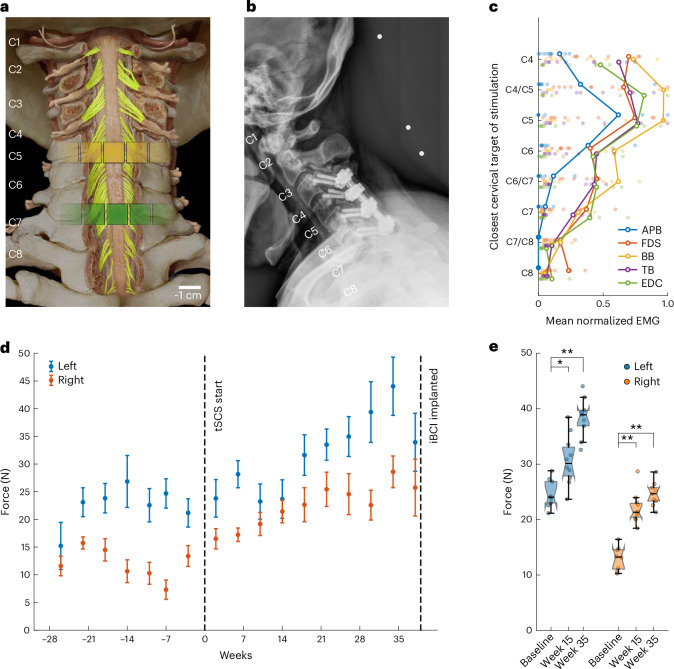
Persistent recovery of upper limb motor function by tSCS. **a**, Schematic showing the locations on the cervical spinal cord dynamically targeted by the custom tSCS electrode patch: C5–C6 (yellow electrodes) and C7–C8 (green electrodes) spinal root. **b**, Cervical spinal X-ray (note the surgical plate and screws for the C3–C6 spinal fusion) and the location of the electrodes. **c**, tSCS-mediated normalized P2P EMG responses averaged across stimulation amplitudes for the muscles of the upper arm. Individual faded circles indicate normalized EMG responses for specific tSCS amplitudes. **d**, Gains in volitional isometric elbow flexion force for the left (blue) and right side (red). Data shown only once in every 4 weeks for clarity. Circles and error bars show mean force and standard deviation, respectively, for one session (*n* = 10 repetitions). **e**, Median gains (central black lines) in isometric elbow flexion force over 15 and 35 weeks after tSCS onset for the left (blue) and right side (red). Boxes around the median show the IQR, notches show the 95% CI and error bars show 1.5 times the IQR. Individual faded circles indicate the forces recorded during the 10-week period centered around the chosen time interval (15 weeks before or 15 and 35 weeks after) from start of tSCS therapy. A one-way repeated measures ANOVA revealed a significant effect of time of measurement for both the right bicep (*F*(2,14) = 41.34, *P* = 1.33 × 10^−6^) and left bicep (*F*(2,16) = 46.49, *P* = 2.16 × 10^−7^) with Tukey–Kramer honestly significant difference multiple-comparison correction showing significant gains at 15 weeks (right bicep: *P* = 0.002, shown with ** and left bicep: *P* = 0.02 shown with *) and at 35 weeks (right bicep: *P* = 0.0003 shown with ** and left bicep: *P* = 3.8 × 10^−5^ shown with **). All data were tested for normality with a Lilliefors test.

For evaluating the efficacy of the hybrid DNB system, we first sought to establish sensorimotor baselines and assess the extent of functional recovery with tSCS alone. The patient presented with minimal ability to lift his arms off his wheelchair armrests, could not bring his hands to his face and had no volitional finger movement. Sensory assessments using Semmes–Weinstein monofilaments (SWMs) confirmed that he lacked tactile sensation in both hands and wrists. We administered tSCS by stimulating the dorsal cervical spinal column via a custom stimulator and an electronically controllable electrode patch^16^ (Fig. 2a) with anatomical landmarks ensuring consistent electrode placement (Fig. 2b). Recruitment profile mapping along the rostrocaudal axis was obtained by delivering 3 Hz tSCS through each of the eight electrode rows of the custom patch at different stimulation amplitudes (100–225 mA), while recording the resultant electromyogram (EMG) from five muscles of the arm and hand bilaterally. For each muscle, peak-to-peak (P2P) amplitudes were normalized to the maximum EMG response observed across all electrode rows and stimulation amplitudes^16–18^ (Methods). Averaging across stimulation amplitudes, this mapping showed that tSCS targeted at the C5–C6 spinal roots strongly recruited proximal as well as distal upper-extremity muscles (Fig. 2c), but the distal muscles including the flexors (flexor digitorum superficialis, FDS) and extensors (extensor digitorum communis, EDC), were more selectively recruited when stimulation was targeted at the C7–C8 spinal roots (Fig. 2c).

On the basis of the recruitment profile, tSCS targeting specific spinal roots was administered paired with activity-based training. Constant stimulation up to 300 mA (50 Hz) was targeted at the C5–C6 spinal root while performing isometric elbow flexion and extension. Similarly, stimulation was targeted at the C7–C8 spinal roots when the participant attempted to open and close his hands. Before initiating tSCS, assessment of volitional forces for 20 weeks established a stable baseline to preclude spontaneous recovery (forces were recorded every week but are shown once every 4 weeks for clarity; Fig. 2d). We determined the baseline forces as the median of the elbow flexion forces recorded over a 10-week period between 9 and 18 weeks before the start of tSCS therapy (baseline median force before tSCS for the right, 13.25 N, interquartile range (IQR) 10.93–14.62, and for the left, 24.03 N, IQR 22.89–26.96). We observed that, within 15 weeks of administering tSCS alone during activity-based training, there was a significant 61% and 25% increase in volitional force output (median elbow flexion forces over a 10-week period between 11 and 20 weeks after the start of tSCS therapy for the right, 21.32 N, IQR 20.26–23.03, and for the left, 30.12 N, IQR 27.54–33.53; data were tested for normality with a Lilliefors test and one-way repeated measures analysis of variance (ANOVA) with Tukey–Kramer honestly significant difference multiple-comparison correction, *P* = 0.002 and *P* = 0.02, respectively; Fig. 2d,e) during isometric right and left elbow flexion, respectively. Further, over a period of approximately 35 weeks since initiating tSCS, gains in volitional force reached up to 86% and 62%, respectively, compared with pre-tSCS baseline volitional forces (median forces over a 10-week period between 30 and 39 weeks after the start of tSCS therapy for the right, 24.65 N, IQR 23.16–25.92, and for the left, 38.90 N, IQR 36.17–40.30; same test, *P* = 0.0003 and *P* = 3.8 × 10^−5^, respectively; Fig. 2d,e). These gains persisted bilaterally over several months and translated into a functional improvement wherein the participant could now bring both his hands up to his face.

**Extended Data Fig. 2:**
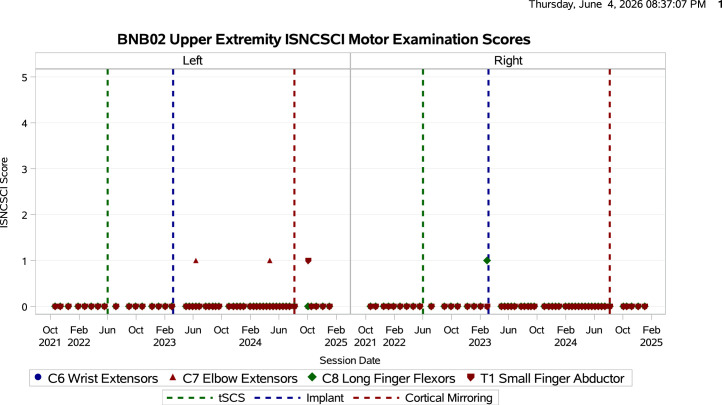
Upper extremity ISNCSCI motor examination scores. Scoring criteria: 0 = Total paralysis; 1 = Palpable or visible contraction; 2 = Active movement, full range of motion (ROM) with gravity eliminated; 3 = Active movement, full ROM against gravity; 4 = Active movement, full ROM against gravity and moderate resistance in a muscle specific position; 5 = (Normal) active movement, full ROM against gravity and full resistance in a functional muscle position expected from an otherwise unimpaired person. ISNCSCI = International Standards for Neurological Classification of Spinal Cord Injury.

However, the therapeutic tSCS targeting the muscles of the hand and triceps did not result in any increase in strength or volitional control (Extended Data Fig. 2). Similarly, we did not observe any improvements in alleviating the tactile sensation deficits in the distal arm. These limitations of administering tSCS alone highlighted the need for a hybrid assistive–therapeutic solution to restore sensory and motor function to the most severely affected regions in complete SCI.

## Restoring cortically mediated hand movement and tactile sensation

**Fig. 3:**
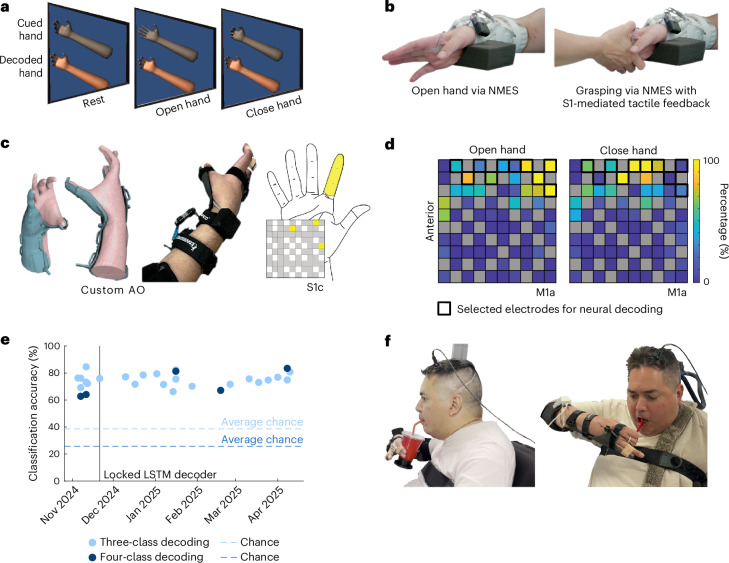
Closed-loop grasping with S1-mediated tactile feedback. **a**, Task design: the participant was instructed to imagine and attempt to rest, close or open his hand by following visual instructions on a screen (gray hand) and to hold the position for variable periods of time. **b**, Targeted activation of the participant’s own hand using cortically mediated NMES to perform open and close movements, in this case, to grasp and feel another person’s hand. **c**, 3D-printed low-profile AO and the sensory percept activated during grasping. **d**, Proportion of sessions in which an electrode was tuned to open the hand versus rest (left) or close the hand versus rest (right), out of 28 sessions (Kruskal–Wallis with post hoc pairwise-comparison Dunnett’s test, *P* < 0.05). Electrodes with a black border were those that had features with an MCC >0.45 in >80% of testing sessions and were selected as input features. **e**, An LSTM decoder was trained on 20 blocks of data and locked. Decoded movements were rest, open, close (three-class) and reaching (four-class). Classification accuracies remained consistently high over >5 months. Decoder performances per session for decoding both three-class (light blue) and four-class (dark blue) movements are shown. **f**, ADLs such as drinking from a cup (left) and self-feeding (right) performed by the participant using both the gains obtained from tSCS therapy and the cortically mediated orthosis.

We next shifted our focus to implementing the assistive portion of the DNB system to address the severely paralyzed hand muscles, and hand/wrist areas void of tactile sensation, that did not improve with tSCS alone. To restore cortically mediated grasping and tactile sensation simultaneously in the participant’s own hand, the DNB system combines real-time M1 decoding and tactile feedback through S1 microstimulation. Two intracortical microelectrode arrays recorded neural activity from the left M1 (Fig. 1c) and drove the activation of the muscles of the participant’s own hand via neuromuscular electrical stimulation (NMES) using a custom-built stimulator and electrode array. Force sensors placed in the palm of the hand provided somatotopically relevant tactile sensory feedback via S1-ICMS (Fig. 1a). The decoder for inferring grasp intent was trained on M1 neural data recorded while the participant attempted to open, close and rest his right hand, guided by a virtual on-screen hand (Fig. 3a). Neural features consisted of the integrated broadband power (100–5,000 Hz) from each electrode computed every 100 ms. We have previously shown that an integrated power band signal allows better decoding performance over time from intracortical arrays, as signal quality of spiking data can fall off rapidly following implantation^19^. For feature selection, only features exhibiting high trial-to-trial consistency in post-cue modulation, quantified by their mean correlation coefficient (MCC; Methods), served as inputs to the decoder. We have previously demonstrated that neural features computed and selected in this manner exhibit highly repeatable amplitude modulation related to movement and tactile stimuli, improving decoding accuracy^19^. For decoding the user’s movement intentions, we specifically chose an LSTM network architecture to address the transient nature of M1 activity patterns^19,20^ and stabilize the decoder output to facilitate sustained hand grasping.

**Extended Data Fig. 3:**
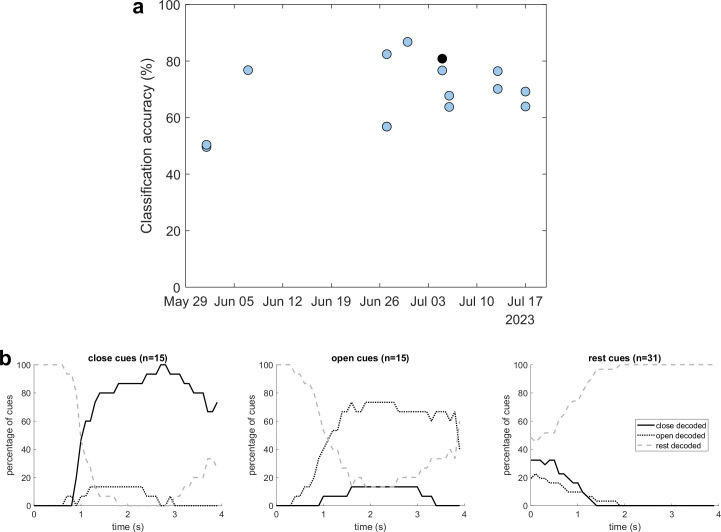
Decoding of attempted hand open and close (see Fig. 3b). **a**) Accuracies for decoding of hand state (close, open, rest) over 15 data blocks collected over 8 session days. Chance = 33.3%. Example data block shown in (**b**) shown in black. **b**) Example decoding performance for one experimental block (overall accuracy=80.8%). For each cue type, over 4 s from the start of that cue, the percentage of trials in each decoder state is shown. The decoder performance could be used to trigger NMES stimulation.

Electrode patches worn on volar and dorsal aspects of the right forearm provided NMES control of finger flexion and extension, respectively. The participant was able to cortically control the muscles of the hand using the LSTM-based decoder (Extended Data Fig. 3), allowing him to open (Fig. 3b, left) and close (Fig. 3b, right) his own physical hand. In one demonstration, he was able to grasp and feel his sister’s hand using his own, otherwise paralyzed and insensate, hand (Supplementary Video 1).

**Extended Data Fig. 4:**
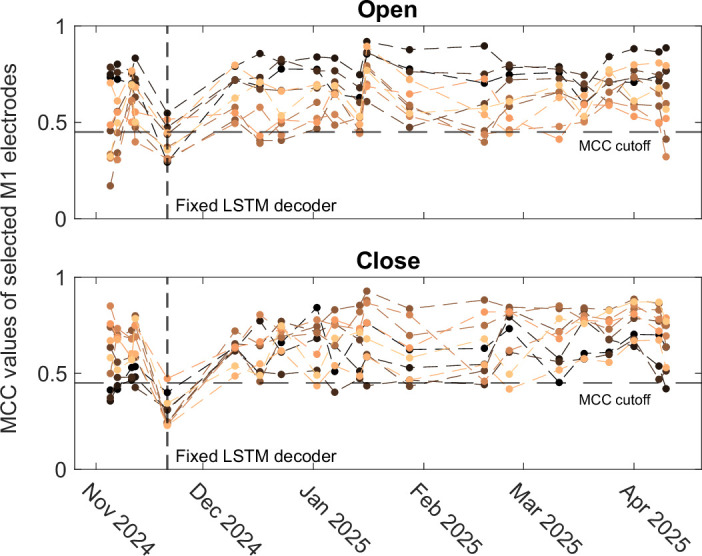
Session-by-session MCC for electrodes selected as inputs for the locked decoder. Dots show the average MCC of a single electrode in a single session (colors identify electrodes). Average MCC was computed over up to the first 2 blocks of the day to allow comparison between session days. Electrodes were selected using the first 20 training sessions if MCC > 0.45 in >80% of those sessions for either class. The vertical dashed line indicates when the decoder was locked; subsequent sessions assess stability only. Selected electrodes exhibit consistently high MCCs across time. Top: MCC when the participant attempted to open their hand. MCC when the participant was attempting to close their hand.

**Extended Data Fig. 5:**
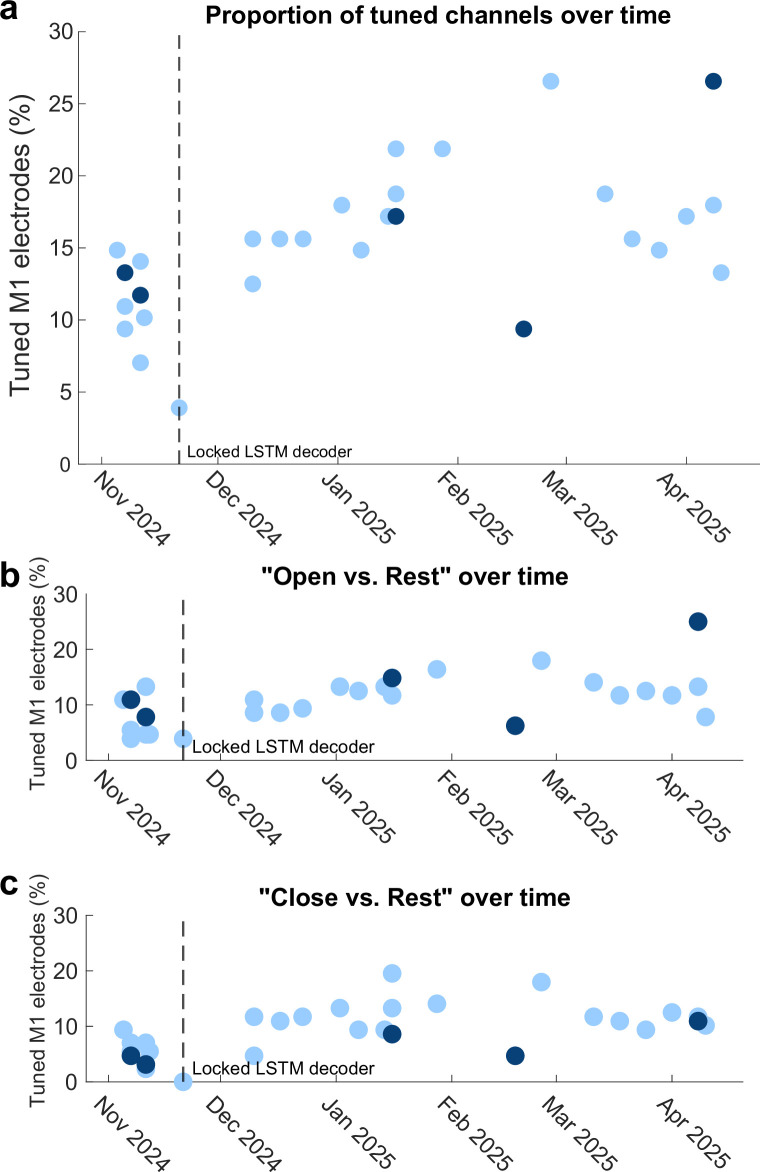
Stability of Electrode Tuning to Movement in M1. Proportion of M1 electrodes exhibiting significant tuning to a cued movement over time, assessed with a Kruskal–Wallis test. For each electrode, neural activity from cue onset to 2 s post-cue was averaged per trial, up to 30 trials per condition per day. Electrodes showing a significant omnibus test (α < 0.05) underwent Dunnett-corrected post hoc comparisons against rest. An electrode was determined to be tuned if at least one movement-rest comparison was significant. Tuning in both 3-class (light blue) and 4-class (dark blue) tasks are shown. **a**): Proportion of electrodes tuned to any condition (“Open vs. Rest” and/or “Close vs. Rest” and/or “Movement vs. Rest”). **b-c**) Proportion of electrodes tuned specifically to “Open hand” (**b**) or “Close hand” (**c**). Vertical dashed line indicates when the LSTM decoder was locked.

**Extended Data Fig. 6:**
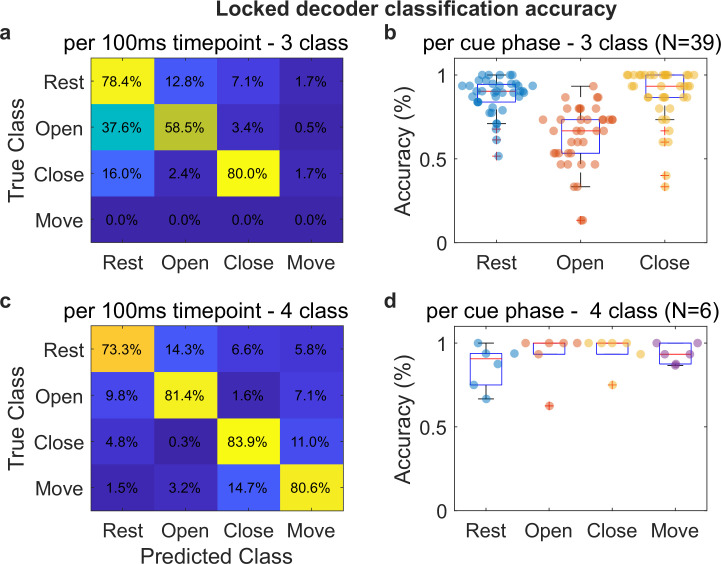
Decoding performance of the stable decoder across all evaluated data blocks. The locked decoder was trained on data blocks containing both 3-class (Rest, Open, Close) and 4-class (Rest, Open, Close, Move) tasks. Performance was assessed in two ways on 3-class data blocks (n = 39) and 4-class data blocks (n = 6). **a**) Confusion matrices showing decoder output per 100-ms time bin, showing the relationship between true and decoded class labels. **b**) Classification accuracy per cue period, where decoding was performed every 100 ms but accuracy was determined by taking the mode (most frequent decoded class) across the entire cue period (3-class: 2-10.5 s; 4-class: 3-13 s). Each dot represents an individual data block, and boxplots summarize variability across blocks for each cue label; center lines indicate medians, box bounds indicate the 25th and 75th percentiles, whiskers indicate the most extreme non-outlier values, and outliers are defined as values more than 1.5xIQR below Q1 or above Q3. **c**) same as a, but for 4-class tasks. **d**) Same as **b**), but for 4-class tasks.

While NMES was able to partially activate certain flexor muscles of the hand, others did not respond, which combined with nerve conduction study results suggested lower motor neuron dysfunction. This made the grasping of objects used in activities of daily living (ADLs) a challenge. To increase grasping forces, we developed a custom 3D-printed low-profile active orthosis (AO) for his right hand that flexed his index and middle fingers using an artificial tendon system for naturalistic grasping. A force sensor embedded in the AO measured grasp strength, which was conveyed to the participant in real-time through multi-electrode S1-ICMS (Fig. 3c; Methods). We used multi-electrode stimulation because it improved detection^10^, as percepts evoked by individual electrode stimulation were reported to be generally weak in perceived intensity. Decoded motor intent activated the AO, which achieved either hand-open or hand-close positions. To further advance the DNB system for real-world use, we aimed to develop a ‘locked’ neural decoder that could sustain a high degree of decoding accuracy without requiring frequent retraining. Training data were collected across six sessions over a 16-day period, capturing neural data for variable durations of rest, hand opening, hand closing (three-class tasks) and reaching movements (four-class tasks), while the participant received real-time feedback from a preliminary decoder. To ensure long-term stability and improved decoder performance^19^, input features were restricted to those that had an MCC >0.45 in >80% of training sessions, yielding a pool of ten M1 electrodes with highly consistent features for the movements of interest. These features showed significant tuning for open or close movements across sessions (Fig. 3d, left: tuning for hand open versus rest; right: tuning for hand close versus rest; Kruskal–Wallis with post hoc Dunnett’s test, *P* < 0.05; Extended Data Fig. 4). A multiclass decoder was trained on these data and then locked, with no retraining. Thus, the same pool of ten electrodes provided the input features for the entire evaluation period (Extended Data Fig. 5). Remarkably, the locked decoder demonstrated consistent performance across all subsequent experiments over a period of 5 months, achieving decoding accuracies up to 84.6% (Fig. 3e and Extended Data Fig. 6). Finally, we evaluated the usability of the DNB system in purposefully designed ADLs, including drinking from a cup and self-feeding. Building on the proximal motor gains achieved through tSCS therapy alone, the participant was then able to transport these objects to the mouth, enabling the successful completion of functional drinking and self-feeding tasks (Fig. 3f and Supplementary Video 2).

## High-precision grasping with deep RL

**Fig. 4:**
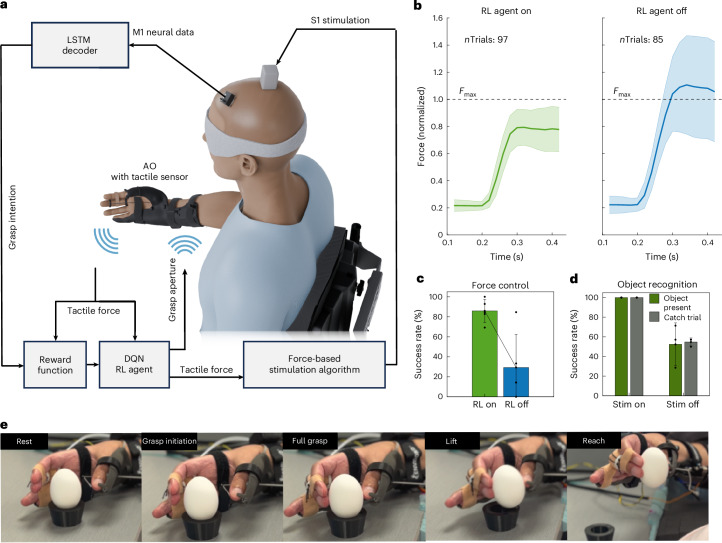
High-precision grasping with tactile feedback. **a**, Experimental setup of the nested neural control architecture using RL for closed-loop grasping. **b**, Mean normalized force across aligned trials; shading indicates standard deviation (*n*Trials = 97 RL-agent-on trials; *n*Trials = 85 RL-agent-off trials). **c**, Successful force control was achieved if the measured force remained under the instructed maximum force level. Bars show mean session-level success rate, error bars indicate standard deviation and dots show individual sessions (*n* = 5 sessions per condition; RL agent on, 87% success; RL agent off, 27% success). Comparison by two-sided paired *t*-test (*P* = 0.012). **d**, The participant achieved successful object presence recognition by lifting the hollow eggshell only on determining that an object was present. Chance, 50%. Bars show percentage correct, error bars indicate standard deviation and dots show individual blocks (*n* = 4 S1-stimulation-on blocks; *n* = 3 S1-stimulation-off blocks). Comparisons used two-sided two-sample *t*-tests (100 g: *P* = 0.0063; 200 g: *P* = 3.1 × 10^−6^). **e**, Demonstration of precision grasp and lift of a hollow eggshell using the AO.

**Extended Data Fig. 7:**
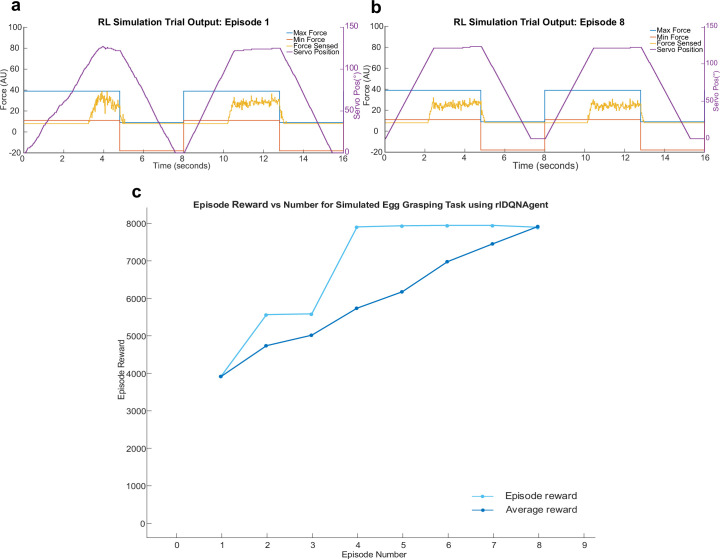
Learning trajectory of the RL algorithm demonstrates rapid convergence across training episodes. **a-b**) RL simulation output showing how the RL agent is able to effectively control the servo motor output (purple) based on force sensor output (yellow) in training episode 1 and training episode 8 respectively. The RL agent is modulating the servo angle to keep the force output within the upper (blue) and lower (red) force bounds **c**) Training of the deep network reinforcement learning agent in 8 episodes showing increasing accruement of rewards.

We observed that LSTM-based decoding of motor intent linked directly to AO control led to poor real-time grasp force regulation leading to under- or oversqueezing of objects. We also previously observed that M1 modulation is mostly transient where the strongest modulation occurs at initiation and termination of movement^19^. Inspired by the hierarchical nature of primate sensorimotor feedback loops^21^, we developed a nested neural network control approach to decouple grasp initiation and continued fine regulation (Fig. 4a). In this approach, while the LSTM decoder inferred grasp intent from M1 neural activity, a deep-RL agent rapidly and continuously regulated grasping forces within specified limits. RL agents typically require thousands of episodes to train in robotic or prosthetic hand-movement applications, which cannot usually be afforded in an iBCI application. Also, given our eventual goal of allowing user-in-the-loop RL training and adaptation, we elected to design an RL agent with discretized states and reward functions to minimize the search space, reducing the training episodes required by several orders of magnitude (Extended Data Fig. 7). The deep-RL agent was trained in a simulated environment where the physical properties of the hand–object interactions were modeled numerically and was rewarded when forces were regulated tightly and responses were faster (Methods). This novel RL-based framework also supports adaptation and future learning as the user interacts with novel objects and refines grasping functionality.

To test the nested LSTM–deep-RL approach, we set up a task involving hollow eggshells (Supplementary Video 3). The participant was blinded to the eggshell and his hand but could see a real-time plot of the grasp force and was instructed to maintain it within a predefined limit. When the deep-RL agent was used, he was able to grasp the eggshell with consistent and safe force levels (Fig. 4b, left). Without the deep-RL agent, the force generated was highly variable and often higher than the predefined limit, *F*_max_ (Fig. 4b, right). The deep-RL agent permitted a significantly higher number of successful trials compared with without it (87% success with RL and 27% success without RL; *P* = 0.012, unpaired *t*-test), demonstrating higher successful force control (Fig. 4c, left). Lastly, the deep-RL approach was used with and without S1-ICMS-based somatosensory feedback, to indicate if the eggshell was within the participant’s grasp. Visually blinded in both cases, the participant achieved a higher success rate (100% versus chance-level success without feedback) when the S1-ICMS-based somatosensory feedback was used, only lifting his arm when he felt he had the eggshell in his grasp (Fig. 4d).

## Persistent recovery of somatosensation

**Fig. 5:**
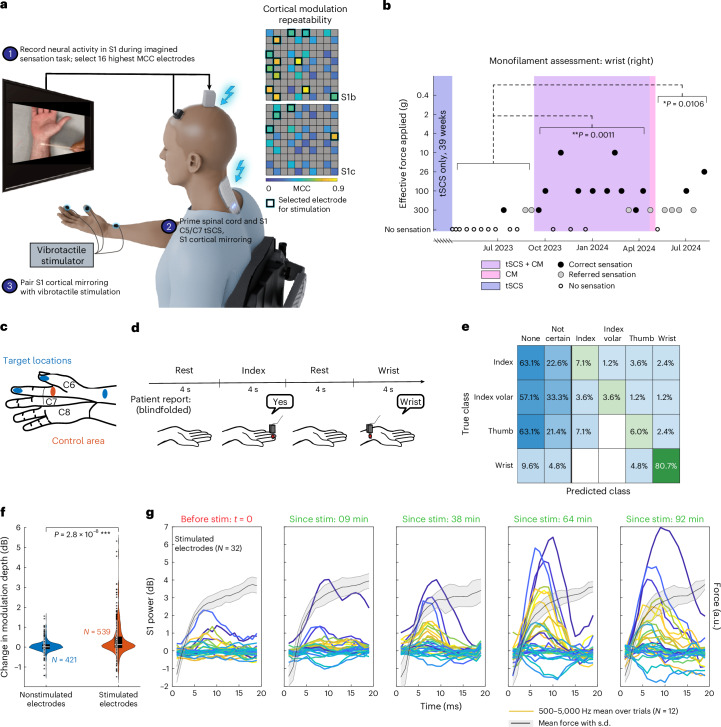
Persistent recovery of somatosensation. **a**, Experimental setup of intervention for persistent recovery of somatosensation. **b**, Sensory assessment results of the right radial wrist over time. Dots represent the lowest effective force applied with a monofilament for correctly localized sensations (black), mislocalized sensations (gray) or when participant reported no sensation (open). Dots show individual assessments (*n* = 13 baseline, *n* = 11 intervention, *n* = 7 post-intervention). Comparisons used two-sided Wilcoxon rank-sum tests with no multiple-comparison adjustment (baseline versus intervention, *P* = 0.0011; baseline versus post-intervention, *P* = 0.0106; intervention versus post-intervention, *P* = 0.2925). **c**, Target locations for intervention: thumb pad, index pad, wrist pad and index volar pad. **d**, ‘Press and hold’ experimental design timeline. In total, 700 g of pressure was applied to the blindfolded participant’s target locations, in 4 randomized order target locations (12 times each). After the touch, the correct location, ‘none’ for a lack of sensation or ‘yes’ for felt but uncertain of the locations, was reported. **e**, Behavioral report confusion matrix for the ‘press and hold’ task over 11 session days. **f**, Violin plots showing the change in depth of modulation before and after tSCS + CM intervention for nonstimulated (blue) and stimulated (orange) electrodes over 10 sessions (*n* = 960; 421 stimulated, 539 nonstimulated). Points represent individual electrode observations; violins show kernel density estimates. The box center lines indicate medians, box bounds indicate the 25th and 75th percentiles and whiskers indicate the minimum and maximum nonoutlier values. Outliers are defined as values more than 1.5 × IQR below Q1 or above Q3. A two-sided linear mixed-effects model with random intercepts for session and electrode ID revealed a significant increase in modulation depth following stimulation (*β* = 0.20, 95% CI 0.13–0.27, *P* = 2.8 × 10^−8^). **g**, An exemplary dataset to show the effect of time since stimulation on integrated broadband power among stimulated electrodes (*N* = 32 total across all 4 target touch locations with each electrode given a unique color for clarity). Neural data were aligned to force onset for each wrist-pressure trial. The gray force trace shows mean ± s.d. across 12 wrist-pressure trials. Neural traces show average neural modulation across the same 12 trials for each stimulated electrode. Average electrode activity was plotted for 0.2 s before and 1.6 s after force onset. Maximum feature amplitude in stimulated electrodes was observed 92 min after the first stimulation was administered.

**Extended Data Fig. 8:**
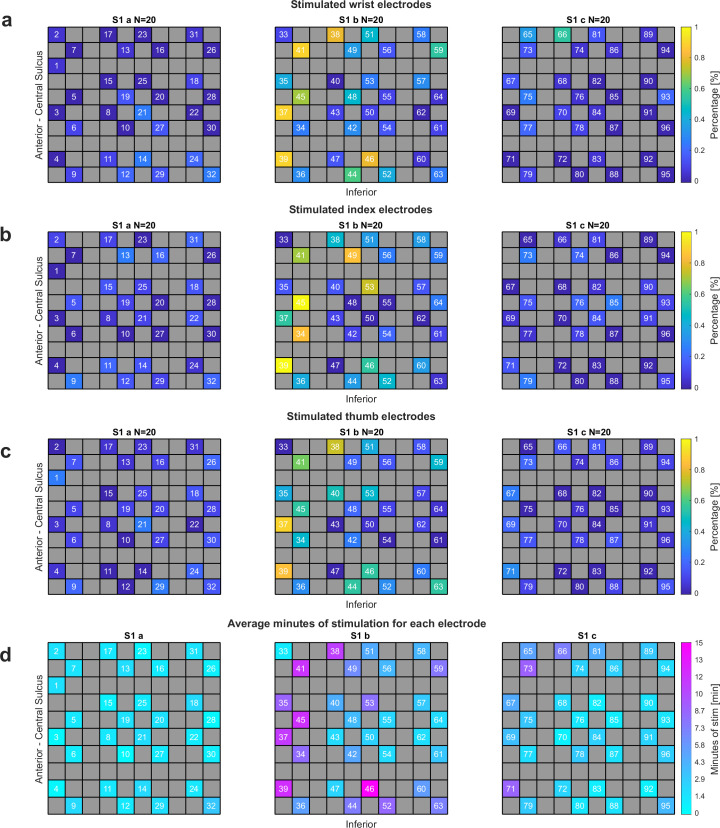
Selected electrodes for cortical mirroring. **a-c**) Proportion of sessions in which each S1 electrode was selected for CM stimulation for the (**a**) wrist, (**b**) index pad, and (**c**) thumb pad target locations computed over 20 sessions. **d**) Average stimulation time (in minutes) per session for each electrode, reflecting total stimulation throughout the entire session day, not limited to hand location.

We next aimed to leverage the therapeutic modality of the DNB to persistently restore tactile sensation in the distal C6–C7 dermatome, specifically targeting the index finger and thumb of the hand as well as the radial wrist, as the previous 39-week tSCS-only regimen had not led to any change. It has been previously shown that pairing of sensory stimulus with neuron-level modulation can induce input threshold changes in the cortex^22^. We explored whether the DNB system could record and deliver patterned cortical stimulation in conjunction with tSCS and peripheral stimulation to promote functional plasticity and recovery of distal somatosensation. To inform the cortical patterns, we developed an intervention termed ‘cortical mirroring’ (CM), which involved delivering stimulation to S1 with spatial patterns that ‘mirrored’ those of the neural activity recorded from the same area during imagined or physical touch. We identified spatiotemporal patterns of cortical modulation in contralateral S1 during imagined, and later, during physical tactile stimuli in the hand and wrist. We reasoned that repeatedly delivering this patterned activation of S1 with peripheral stimuli, in conjunction with a tSCS-mediated increase in spinal circuitry excitability^23^ (tSCS + CM), could promote short and/or long-term plasticity. During the tSCS + CM intervention, the participant engaged in sensorimotor imagery tasks aimed at enhancing impaired sensorimotor processes (Fig. 5a). As the first step in the CM approach, neural activity was recorded while the participant watched hand/wrist stroking and imagined corresponding sensations on four different locations on his right hand: index pad, index volar pad, thumb pad and wrist. Next, we analyzed the integrated broadband power (100–5,000 Hz) for each of the 96 electrodes in S1 and identified up to 16 electrodes (per each hand/wrist location) that had the most consistently modulated power (highest MCC >0.4). Across 20 sessions, we observed consistency in electrodes with neural features that repeatedly exhibited high MCC, and at least 50 % of the electrodes chosen for CM stimulation (for each tactile area) were used in more than 50% of the sessions (Extended Data Fig. 8). However, to permit plasticity over time, we continuously updated the population of electrodes used for CM stimulation every session to reflect the most recent state of the cortical circuitry. Subsequently, during CM, we delivered high-frequency (200 Hz) modulated stimulation (5 s on–5 s off) on these chosen electrodes (up to the maximum possible of 16 electrodes simultaneously owing to hardware limitations) mirroring the natural activation of S1 observed during imagined sensation. This was paired with vibrotactile stimulation for up to 10 min. The index volar pad location on the hand served as a control, and no vibrotactile stimuli or CM/S1 stimulation was performed related to this location.

We observed that tactile sensitivity, as assessed using SWMs on the radial aspect of the palmar side of the right wrist, significantly improved during the tSCS + CM intervention period (Fig. 5b). Here, the blindfolded participant had a significantly lower threshold of correctly perceiving and localizing the sensation evoked by the monofilaments during the intervention period compared with the baseline period (Fig. 5b, black dots signifying ‘correct’ perception; Wilcoxon rank sum test on the scores awarded on the basis of the thinnest filament perceived, *P* = 0.0011; Methods), perceiving as low as 10 g of effective applied force on the wrist. The post-intervention period presented significantly more instances where the participant perceived the sensation but could not localize it correctly (Fig. 5b, gray dots signifying ‘mislocalized’ perceptions; Wilcoxon rank sum test, *P* = 0.0106). The participant reported ‘no sensation’ perceived at the index pad, index volar pad and thumb pad bilaterally across all sessions across all experimental phases. These results suggest that the tSCS + CM intervention induced long-term plasticity changes in wrist sensitivity.

To study the effect of the intervention (tSCS + CM) on S1 modulation during tactile stimuli, a ‘press and hold’ task was developed. Here, the blindfolded participant’s neural and verbal feedback responses were evaluated while an experimenter administered 700 g of force to each of the four previously described hand locations using a handheld force sensor (Fig. 5c). The participant indicated verbally whether he felt the sensation and the perceived location of the sensation (Fig. 5d). The participant could feel his wrist with a high average accuracy of 81%, in contrast to other locations, over 11 sessions (Fig. 5e).

**Extended Data Fig. 9:**
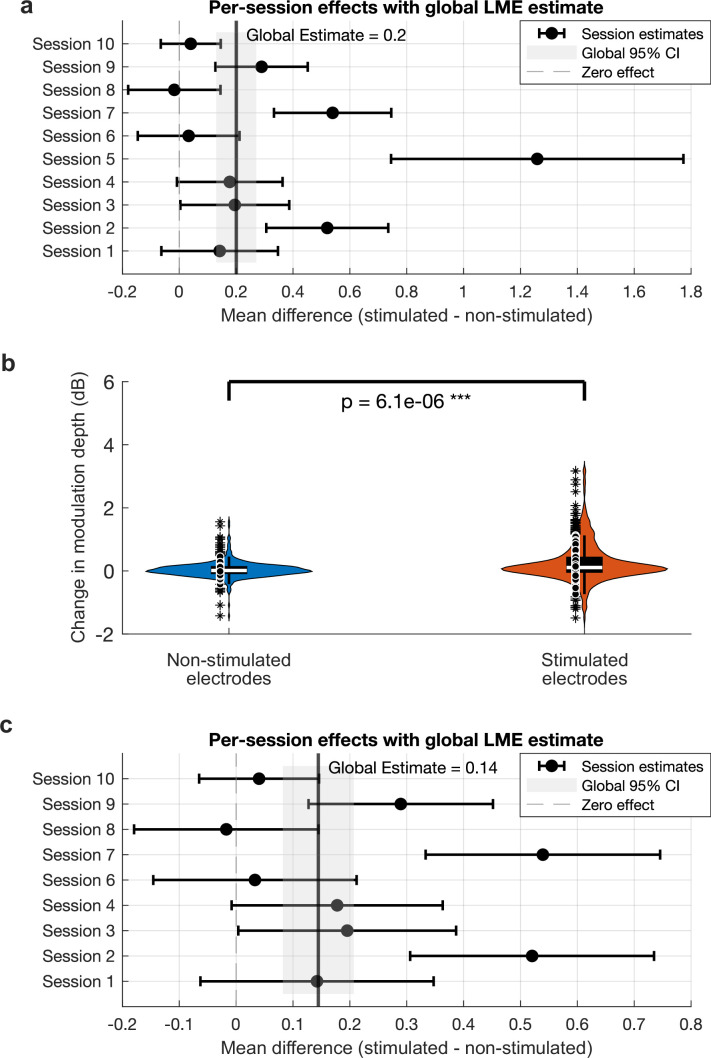
Effects of stimulation on modulation depth. **a**) Per-session stimulation effects, plotted as the difference in mean modulation change between stimulated (total n = 421) and non-stimulated (total n = 539) electrodes for each session (n = 10 sessions; n = 96 electrodes per session, per-session n = 28-59 stimulated and n = 37-68 non-stimulated electrodes). Points indicate session-level estimates and error bars indicate 95% confidence intervals. The vertical line and shaded band indicate the global fixed-effect estimate and its 95% confidence interval from a two-sided linear mixed-effects model with random intercepts for session and electrode ID (beta = 0.20, 95% CI [0.13, 0.27], p = 2.8 × 10^−8^). **b**) Changes in modulation depth after removal of the outlier session from panel a (session 5), comparing non-stimulated (n Total=475) and stimulated (n Total=389) electrodes. Points represent individual electrode observations; violins show kernel density estimates. Box center lines indicate medians, box bounds indicate the 25th and 75th percentiles, and whiskers indicate the minimum and maximum non-outlier values. Outliers are defined as values more than 1.5xIQR below Q1 or above Q3. A two-sided linear mixed-effects model with random intercepts for session and electrode ID revealed a significant increase in modulation depth following stimulation (β = 0.14, 95% CI [0.08, 0.21], p = 6.1 × 10^−6). **c**) Same as panel a, but with session 5 removed (n = 9 sessions; n = 864). The same two-sided linear mixed-effects model estimated a stimulation effect of beta = 0.14, 95% CI [0.08, 0.21], p = 6.1 x 10^−6^.

**Extended Data Fig. 10:**
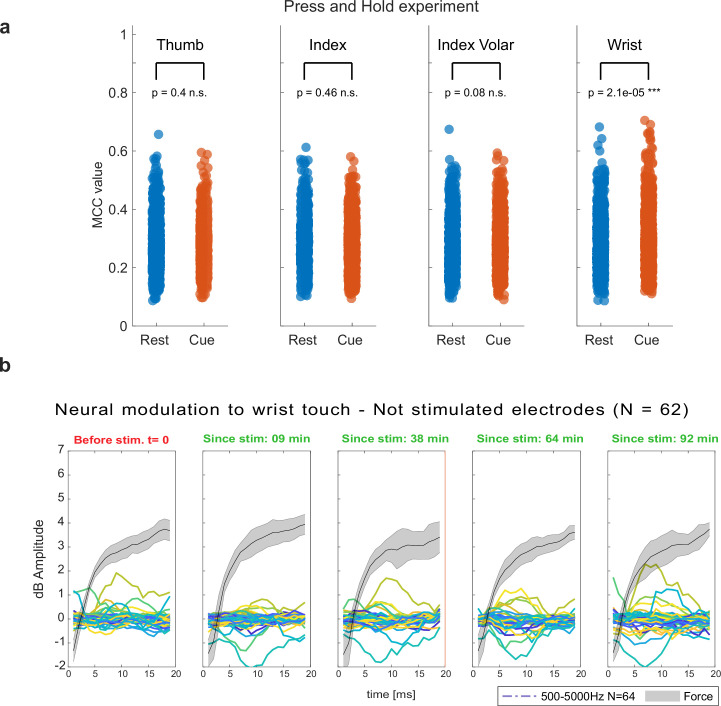
Neural modulation to Press and Hold task. **a**) MCC comparison of 960 electrodes (96 electrodes x 10 sessions) during a 1.4-s rest period versus a 1.4-s period during applied pressure to the thumb pad, index pad, index volar pad and wrist. Comparisons used two-sided paired t-tests for rest versus pressure-cue MCC values at each location. Only the wrist demonstrated a significant difference in activity (thumb pad, p = 0.40; index pad, p = 0.46; index volar pad, p = 0.080; wrist, p = 2.1 x 10^−5^). **b**) Effect of time since stimulation on feature amplitude in non-stimulated electrodes (N = 64, one color per electrode). Neural data were aligned to force onset for each wrist-pressure trial. Force traces show mean +/- standard deviation across 12 wrist-pressure trials within each block. Neural traces show the average neural modulation across the same 12 trials for each electrode. Average electrode activity was plotted for 0.2 s before and 1.6 s after force onset.

Neural activity recorded in S1 during this task showed correspondence with the monofilament (Fig. 5b) and behavioral results (Fig. 5e), with significantly higher MCC values for physical stimuli (with the force sensor) compared with visual stimuli alone (paired *t*-test, *P* = 2.1 × 10^−5^), but only for the wrist location (Extended Data Fig. 10a). Interestingly, all S1 electrodes involved in CM for different hand locations when taken together had significantly increased modulation depth (using a linear mixed-effects model) to physical stimuli after the tSCS + CM intervention, in contrast to nonstimulated electrodes (Fig. 5f, unpaired *t*-test, *P* = 2.8 × 10^−8^; Extended Data Fig. 9). Importantly, neural activity recorded during the ‘press and hold’ task exhibited a continued increase in modulation for an integrated broadband power sub-band (500–5,000 Hz) over a 92-min period after stimulation ceased (Fig. 5g). These striking progressive increases in modulation were absent in nonstimulated electrodes (Extended Data Fig. 10b), suggesting that short-term, electrode-specific plasticity effects potentiated the wrist sensitivity improvement.

## Discussion

Paralysis can drastically impair independence and quality of life. For those living with tetraplegia, regaining hand function is a top priority, above walking, bowel, bladder or sexual function^24^, with extremely limited options for functional recovery. For this important clinical need, we conceived, developed and demonstrated a DNB, a hybrid assistive–therapeutic system that integrates an iBCI, NMES and AO, and patterned cortical and spinal cord neuromodulation to acutely and persistently restore hand movement and tactile sensation in complete tetraplegia.

Our work and those by others have shown that functional recovery following SCS is restricted to muscles that show some level of residual volitional control^16,25,26^. Recent work suggests that tonic SCS renders residual supraspinal input functionally effective by altering motor recruitment thresholds, in a manner constrained by lesion severity^27^. In this work, we demonstrate that the therapeutic module of the DNB system, focusing on spinal cord neuromodulation, could persistently improve the volitional control of proximal arm function. Meanwhile, the assistive module of the DNB system could provide iBCI-mediated volitional control and real-time tactile feedback to the user’s own hand. When combined, the hybrid assistive–therapeutic DNB system enabled independent performance of ADLs, including self-feeding and drinking, as well as meaningful tactile interaction with a loved one’s hand. Under controlled conditions, the participant further demonstrated iBCI-mediated high-precision grasping, successfully manipulating fragile hollow eggshells in real time.

Outside of study sessions, the participant self-reported the ability to independently scratch and wipe his face. Notably, he has reported being able to pet his dog and feel her fur as well on his previously insensate right wrist. We believe these anecdotal gains in function further reflect the persistent restoration of function from the DNB system, specifically the significant increase in bicep strength and the improvement in sensitivity at the wrist as observed in the SWM sensory test.

Using an LSTM decoder, we were able to decode four distinct hand and arm states, including rest, hand opening, hand closing and reaching. Although M1 neural recordings were obtained from 128 electrodes, the motor decoding described here was achieved using the features from only ten electrodes identified through MCC-based feature selection. This selection prioritized electrodes with features that demonstrated consistent task-related modulation across the training period, and as we have shown previously, can improve decoder accuracy^19^. Such a selective input feature set enabled the LSTM decoder to achieve consistently high levels of decoding accuracy over several months without retraining. While a larger proportion of electrodes potentially showed task-related tuning on any given day, they did not maintain stable tuning over time and, hence, were excluded. Inclusion of such transiently tuned electrodes could negatively affect decoder performance over time. Although the degrees of freedom are fewer than what other iBCI systems have demonstrated, this present work is proof of principle in low-latency, stable decoding of functional movements to enable ADLs. In addition, it is highly likely that these excluded electrodes stably encode additional degrees of freedom of movement and could be included as the decoder is trained for additional movements. Thus, with the LSTM-based architecture for the decoder and MCC-based feature selection, we were able to address the transient nature of motor activity, maintain performance accuracy over extended periods of time^28^ and achieve reliable decoding of grasp intent from M1 activity^29^.

One of the challenges of restoring iBCI-mediated motor control in the human hand has been achieving precise grasp regulation for real-world tasks, such as handling fragile objects and foods. Using a novel deep-RL approach, we demonstrated a proof-of-concept system capable of rapidly and accurately stabilizing grasp force, enabling successful interactions with delicate objects. Inspired by the distributed neural network architecture in primates^30^, we developed a nested control architecture combining a highly stable recurrent neural network for motor intent decoding and deep RL for force stabilization to allow the participant to perform precision-grasping tasks with high success. This system enabled the participant to complete the task even while conversing with people simultaneously, suggesting reduced cognitive load and future applicability of this approach in real-world settings. One of the limitations of the current work is that the task required achieving only a single target force while attempting to grasp a single type of object. However, this framework can be extended to accommodate multiple objects that require varying grasp forces. It has been demonstrated that LSTM-mediated decoding of multiple force levels from M1 recordings is possible^29^. Within our architecture, following the decoding of force by the LSTM decoder, the nested-RL network would act to rapidly stabilize the applied force within tight bounds around the decoded or desired target, enabling adaptive, object-specific grasp regulation.

In addition to motor improvements, we leveraged the benefits of the modular and bidirectional nature of the DNB system, combining recording and stimulation capabilities with the assistive and therapeutic modalities for somatosensory recovery. In this set of experiments, CM approximated the circuit-level activity observed during imagined sensation. Pairing this with tSCS and peripheral stimuli resulted in persistent recovery of somatosensation that was functional even when the DNB was out of the loop in an individual with a sensory complete SCI. tSCS has been previously demonstrated to increase the excitability of the spinal circuitry^23^ and has also contributed to persistent sensory restoration^16,31^. We hypothesized that tSCS could facilitate the resting potential of the interneuronal networks of the spinal cord allowing transmission of peripheral tactile afferents, attenuated by the injury, to the brain and aid in the CM paradigm. This intervention enabled the participant, with impaired sensation in his right wrist, to detect significantly lower force levels, gains that persisted for more than 2 months even after cessation of stimulation in a follow-up period. It has been observed that repeated high-frequency stimulation at the periphery^32^ or repeated electrical stimulation of cortical ensembles^33^ can potentiate cortical responses to incoming stimulus such as touch. We found that neural modulation of stimulated electrodes to physical touches increased after S1 CM, which is consistent with spike-triggered microstimulation pairing results in primates^33^. While the vibrotactile stimulation was optimized for the peripheral receptors at 120 Hz, the cortical stimulation was at a much higher frequency of 200 Hz, compared with the microstimulation pairing study in primates^33^, as this has been shown to efficiently induce plasticity in networks, not just individual neurons^34^. Our aim was to activate the circuitry at a network level to promote neuroplasticity instead of focusing on intracortical neuronal pairings. This electrode-specific increase of neural modulation suggests that intervention-dependent cortical plasticity may have contributed to sensitivity improvement. Touches at other locations on the hand neither caused S1 neural modulation before or after intervention nor did they improve over time. This suggests a minimum level of residual signal transmission via the spinal cord to S1 may be necessary to allow intervention-dependent sensory improvements, as observed for motor rehabilitation^26^. It has been shown that vision can modulate the S1 area but in a much stronger fashion if there is an associated physical element^35^. It could be interesting to test if giving the participant visual input during CM and paired physical stimulation plays a critical role in improving the functional gains. iBCIs have made tremendous progress in restoring the sense of touch acutely as robotic or virtual hands interact with objects^6,11^. Also, recent studies have shown that modifying stimulation parameters to provide biomimetic stimulation improves sensory detection and performance in humans^10^. Such biomimetic stimulation entails temporal patterning of stimulation with the aim of activating cortical neurons in a more naturalistic manner to improve perception of acutely restored artificial sensations. Biomimetic stimulation is usually delivered through either one or a few electrodes spatially clustered within the target receptive field of the percept. CM, however, is designed to recreate the broader network-level activity observed during natural touch to raise excitability and facilitate neuroplasticity. This might even include neurons not directly involved in touch perception but engaged in relevant adjacent processes. While distinct in their design and aims, the two paradigms could be combined to leverage their unique advantages. For instance, CM incorporating finer temporal characteristics, as designed in biomimetic stimulation, could be even more effective in evoking neuroplasticity by activating the relevant cortical circuitry with more natural modulation patterns.

Although evaluated in a single participant, this study establishes feasibility and highlights the potential functional benefits of such a hybrid DNB system in complete SCI. Scaling the DNB system to a broader population will require addressing multiple challenges such as optimizing tSCS mapping and stimulation parameters, adapting of the iBCI decoder and the RL system to interindividual variability and streamlined or automatic identification of effective spatiotemporal stimulation patterns for CM. However, the modular design of the DNB system makes it adaptable to the specific needs of the participant dictated by injury and residual function. The choice of a transcutaneous approach to spinal cord and peripheral neuromodulation renders the DNB more amenable to clinical translation. While epidural or percutaneous approaches afford more selective stimulation, they potentially carry a higher surgical risk burden, especially when targeting the cervical spinal cord in SCI. Similarly, adapting to diverse neurological conditions, including stroke, is more feasible with the reduced surgical burden compared with epidural SCS as recently demonstrated^36^. Nevertheless, it would require careful evaluation of condition-specific factors and the risk–benefit profile when considering intracortical implantation^37^.

The DNB system, although modular and adaptable, is still a highly specialized system requiring a team of highly trained personnel to operate. The RL and wearable tSCS systems could make home use easier, and other studies have already shown improved home-use scenarios for implanted BCIs. Also, observed clinical benefits with just two sessions per week, which is fewer than other tSCS studies (typically three to five times per week) thereby making it more amenable to home use.

In this work, the assistive and therapeutic aspects of the hybrid DNB system facilitated both in-use and after-use functional abilities. By restoring persistent motor and sensory gains, the therapeutic modalities of the DNB can provide continued benefits to the user even when not actively using the system. Restoring movement and sensation in the participant’s own hand allowed him to perform ADLs and have increased independence, while enabling socially and emotionally rewarding engagements. Together, these results demonstrate that a hybrid neuroprosthetic–neuromodulatory DNB can engage brain and spinal pathways to enable functional and precision grasping, while supporting persistent sensorimotor recovery in complete SCI.

## Methods

### Study design and participant

The study is registered at ClinicalTrials.gov (NCT03680872) and was conducted under an Investigational Device Exemption (IDE G170200) issued by the US Food and Drug Administration (FDA). The protocol was approved by the Northwell Health Institutional Review Board (IRB no. 17-0840) and was in accordance with the Declaration of Helsinki. The participant provided written informed consent for study participation and permission for the use of photos, along with audio and video recordings, in which he appears.

The participant (male, 42-years old at study enrollment) sustained a bilateral C4 sensory/C5 motor American Spinal Injury Association (ASIA) Impairment Scale A SCI following a diving accident in 2020. He underwent a C3–C6 spinal fusion. At the time of enrollment, it was 13 months after the participant had sustained the injury and he had undergone physiotherapy and required complete assistance with all ADLs. The study involved different phases shown in Extended Data Fig. 1. For the first year of the study, the participant underwent activity-based training paired with tSCS once per week. Surgical planning was performed using a 3 T magnetic resonance imaging (MRI) scanner followed by implantation of an iBCI, and the study participant attended experimental sessions one to three times per week.

### Upper-extremity motor and sensory clinical assessments

Upper-extremity strength and sensation were assessed every 2 weeks. Strength was assessed using the motor examination for upper extremities protocol established by the International Standards for Neurological Classification of Spinal Cord Injury (ISNCSCI). Tactile sensation was evaluated using SWMs (North Coast Medical) designated as 6.65f, 6.10f, 5.46f, 5.07f, 4.56f, 4.31f and 3.61f, corresponding to the logarithm of effective applied force of 300, 100, 26, 10, 4, 2 and 0.40 g, respectively. The participant, while blindfolded, reported the perceived location of three 1.5-s taps per filament applied in descending order of effective force (filament thickness) to randomized hand/wrist locations with no intervening washout periods. ‘Correct’ indicated accurate localization of perceived sensation, ‘no sensation’ indicated absence of perception, while ‘mislocalized sensation’ indicated a mislocalized perception. Starting with the 6.65f filament, testing stopped at the first absent or mislocalized perception. Scoring was based on the thinnest monofilament perceived; with no sensation scored as 0, mislocalized sensation as 0.5 and each correctly perceived and localized filament scored from 6.65 f = 1 through 3.61f = 7 (ref. ^38^). Changes in perceived sensations over experimental time periods were assessed using Wilcoxon rank sum tests.

### Activity-based training involving elbow extension and flexion

The general activity-based training protocol has been previously published^16^ and is briefly summarized here. The participant’s arm was placed on a custom-built board fixed with a tension/compression load cell (25 lb, Model M31, Honeywell International), with the wrist strapped over the load cell to permit isometric force measurement of elbow flexion and extension. The arm was extended forward and elbow flexed at 10° and 15° for flexion and extension tasks, respectively. EMG activity and force were recorded for each task through Noraxon MyoMotion (Noraxon USA) for abductor pollicis brevis (APB), biceps brachii (BB), triceps brachii (TB), FDS and EDC muscles. Each task consisted of two sets of five 5-s repetitions (flexion or extension) followed by 5 s of rest, with a 3–4-min interset rest. Audio cues signaled the start and end of each repetition. The participant was verbally encouraged and provided a visual force goal to maximize and sustain force generation. All tasks were performed for both arms.

### tSCS

tSCS was delivered using a custom stimulator and an electronically steerable electrode patch^16^. The stimulator consisted of an ESP32 feather microcontroller (Espressif Systems) that digitally generated a biphasic 10-kHz, 0.5-ms-long (five cycles) sinusoidal waveform repeated at 50 Hz. A voltage-driven digital input class-D audio amplifier (TAS5825PEVM, Texas Instruments) scaled the waveform to the desired stimulation amplitude. A 10:1 step-up transformer (42TM003-RC, Xicon) helped to increase voltage output and provided signal isolation and impedance matching. Current limit circuitry was used to ensure that the power density never exceeded 0.25 W cm^−^^2^. The stimulator was powered by a 24-V battery.

Consistent placement of the custom transcutaneous patch was ensured using the inion of the external occipital protuberance as a landmark. Two interconnected 5 × 10-cm hydrogel electrodes (Axelgaard Manufacturing) placed along the lumbar spine midline served as return electrodes (anodes). To target specific cervical segments of the spinal cord, stimulation was provided simultaneously to three adjacent electrodes (electrode dimension, 10 mm × 10 mm, 1 mm pitch) within a single row, spanning the cervical midline.

### tSCS-mediated muscle recruitment

Recruitment profiles were generated by administering tSCS at 3 Hz. Increasing stimulation amplitudes (100–225 mA, in ~25-mA increments) were applied to each of eight electrode rows for up to 15 s while recording EMG from the APB, BB, TB, FDS and EDC muscles bilaterally. For each of the 30–50 stimulation pulse, snippets (5–55 ms after the stimulation pulse) with P2P amplitudes exceeding five times the baseline standard deviation were analyzed for each channel. If the EMG response did not cross threshold, it was recorded as 0. It was recorded as ‘NaN’ if the channel was not recorded or corrupted and these were not included in further analysis. For each muscle, P2P amplitudes, averaged across stimulation pulses, were normalized to the maximum P2P observed for that muscle across all amplitudes and electrode rows. Recruitment curves for each muscle were generated by averaging this normalized P2P across amplitudes that showed any muscle recruitment. An X-ray image in the sagittal plane, with radio-opaque markers placed at 7 cm and 9.2 cm caudal to the participant’s occipital protuberance, was used to determine the electrode location relative to the spinal roots and vertebral landmarks.

### tSCS with activity-based training

The experimental protocol began with 10 min of tSCS priming over the C5–C6 spinal root, during which the participant was asked to rest and not move during this time. This was followed by pseudorandomized left or right elbow flexion and extension tasks (as described above) with tSCS actively stimulating. After this, 10 min of tSCS priming over the C7–C8 spinal root was followed by a bilateral hand open-and-close task wherein the participant is attempting to open and close his hands guided by a virtual hand, with the tSCS actively stimulating.

### fMRI

The participant underwent scanning in a 3 T MRI scanner (Prisma, Siemens) and a 64-channel head–neck coil with HCP-style^39^ structural and functional MRI protocols (for scanning parameters, see Supplementary Table 1). To localize hand representations in the sensorimotor cortex, the participant performed motor- and sensory-specific tasks during fMRI acquisition. Whereas for M1, the task consisted of video-guided imagining and attempting of finger flexion (thumb, index or middle), for S1, the task involved imagining sensation while watching videos of a Q-tip stroking the same fingers. Each task consisted of 12 s of activity (flexion or stroking), followed by 12 s of rest, with ten repetitions. In addition, for S1 tasks, a Transcutaneous Electrical Nerve Stimulation (TENS) unit (LG TecElite) delivered electrical stimulation (up to 10 mA, 30 Hz, 300-µs pulse width) to the same fingers using ring electrodes (Natus Medical) placed across the first knuckle. A programmed microcontroller activated solid state relays to target the correct finger and control the stimulation timing (12 s on, 12 s off) to synchronize with the Q-tip videos. The sensory tasks were performed in three varieties: Q-tip video only, TENS only and both simultaneously. However, the activation observed in S1 was weak and was ultimately not used for array-implantation planning.

MRI preprocessing used the HCP minimal preprocessing pipelines (v3.27 (ref. ^39^)) including motion and distortion correction, cortical surface reconstruction and subcortical segmentation, T1-weighted/T2-weighted-based myelin content and cortical thickness maps, fMRI data transformation to MNI and CIFTI gray-ordinate standard spaces using MSMAll-based registration^40,41^ and 6-mm surface smoothing. Spatially specific structured noise was removed using the HCP’s multirun (v4.0) ICA-FIX^42–44^ with training data for multi-fMRI (multiple finger tasks) and linear trends with a high pass filter (>0.1 Hz) without regressing out motion parameters. Somatotopic functional responses were estimated using a generalized linear model (GLM)-based fMRI analysis^45^ on the gray-ordinate data space for each finger.

Task fMRI activation maps, visualized in Connectome Workbench^15^, guided array implantation. Planned locations were identified as cortical vertices, with 4-mm borders, transformed into volume space regions of interest (ROIs) (AC-PC aligned T1-weighted) (wb_command -surface-geodesic-rois, -metric-label-import, followed by -label-to-volume-mapping). These volumetric ROIs were registered to the native T1-weighted image, thresholded to create binary masks and merged into a single label volume using fslmaths. The merged label was ‘burned’ into the native T1 and exported as a NIfTI file (nii.gz) to ensure compatibility with the surgical navigation system (StealthStation, Medtronic).

### Intraoperative cortical stimulation

Intraoperative motor and sensory mapping were performed via direct cortical surface stimulation using a 2-mm ball tip monopolar or bipolar probe, respectively. Under anesthesia, motor responses were elicited via 3-s-long, 50-Hz, 4-mA, biphasic stimulation trains (pulse width, 100 μs), with high-impedance rejection enabled, and measured using EMG. Stimulation of the superior and inferior M1 elicited EMG responses in the upper arm and frontalis muscles, respectively. Hand twitches in response to motor cortex stimulation were also noted and this location was marked for M1 array implantation. Furthermore, awake positive sensory mapping was performed to determine tactile percepts for various stimulation points in the somatosensory cortex. Four of the fingers (Fig. 1b) were localized successfully and these responses were used to finalize the sites to target for S1 array implantation.

### Array implantation

The participant was implanted with five 10 × 10, 1.5-mm platinum microelectrode arrays with sputtered iridium oxide film (SIROF)-coated tips (Neuroport Array, Blackrock Neurotech) in the hand areas of the M1 (2 × 64-channel arrays, 128 total) and S1 (three sparsely populated arrays, 32 channels each; 96 total) in the left hemisphere. Following M1 mapping under anesthesia and awake positive mapping of the S1, the arrays were implanted using a high-speed pneumatic press. Both M1 arrays were connected to a single pedestal mounted anteriorly, and all S1 arrays were connected to a second pedestal mounted posterior medial to the craniotomy, followed by a complex neuroplastics closure.

### S1 mapping

S1-ICMS through SIROF microelectrode arrays using a Cerestim R96 microstimulator (Blackrock Neurotech) generated percepts at the fingertips of the participant. Sensory stimulation waveforms led with a cathodic phase of 100 µA, pulse width of 300 µs and interphase width of 100 µs. Individual electrodes were stimulated at 50 Hz for 2 s to generate percepts in the palmar side of the index and thumb regions. The zone of perception was drawn on a hand image using a custom Python script, as verbally described by the participant.

### Sensorimotor-task design

A session corresponded to a recording period within a calendar day. All data collected within that day belonged to the same session. A block corresponded to a contiguous run within a session, consisting of multiple trials of a few different movements ordered either randomly or in a specific sequence.

The participant performed an attempted movement task cued by a virtual hand (Unity Software Technologies) to open, close or rest his right hand. The cued followed a 4-s cue (maintain movement), 4-s delay (rest) paradigm. A total of 15 repetitions of each motion (open or close) were performed. Cue orders were randomized.

### Neural preprocessing pipeline

Broadband electrical activity was recorded from the NeuroPort arrays using Neural Signal Processors (Blackrock Microsystems). Analog signals were amplified and digitized at 10 kHz. Nonoverlapping periodic Blackman windows of 1,000 samples (100 ms) were applied to reduce spectral leakage and improve frequency-domain estimation, followed by a short-time Fourier transform (STFT) with 10-Hz frequency resolution. Signal amplitudes were integrated across predefined frequency bands (100–5,000 Hz), smoothed using a 1-s boxcar filter, log-compressed for normalization and drift correction, standardized using a 24-s running mean and scaled to decibels by multiplying by 20. These frequency bands have been shown to exhibit highly repeatable amplitude modulation related to movement and tactile stimuli^19^. For online decoding, the boxcar filter was reduced to 200 ms for a smoother output.

### Feature selection

Feature selection on training blocks used the MCC of each feature. Integrated broadband power, computed every 100 ms for each trial within a temporal window (0.2–1.6 s after cue onset), constituted a feature per electrode. For each of these electrodes, the correlation was computed between the trial-by-trial response and the average response of that feature across trials for each cued movement. Averaging these correlation coefficients across trials gives the MCC for that feature. High MCC values indicate features/electrodes with activity that consistently and repeatably modulates during each trial^19^.

### Locked decoder-training design

Training data were collected over five sessions within a 16-day period using two paradigms.

Experiment 1 (total of 18 data blocks): the participant attempted to move his right hand by mirroring virtual hand movements cued on a screen to open, close and rest his hand for variable durations (rest time, 1.5–11.5 s; open hand, 1.5–4.5 s; close hand, 1.5–5.5 s). Rest, open and close were randomly interleaved; each block contained 15 open and 15 closed trials and a minimum of 15 rest trials (variable based on transitions).

Experiment 2 (four data blocks): the participant was instructed to perform a sequence of cued hand closing, reaching up and down (emulating drinking from a cup) and opening of his right hand for variable durations (rest time, 4–13 s; open hand, 3–6 s; close hand, 3–6 s; reaching, 5–6.5 s). Each block contained 15 repetitions of the fixed sequence.

The decoder was trained on 20 sessions and tested on 2 (1 of each experimental type). The first nine data blocks were recorded without feedback (offline). The subsequent 11 blocks included real-time decoding feedback of decoded hand movements in the form of a second hand on the screen. LSTM models were trained iteratively as more data became available.

Out of 22 blocks, 20 trained the final decoder and 2 (1 per experiment) tested the decoder. Only M1 data were used, as S1 was used for stimulation. Electrodes with an MCC >0.45 in >80% of training blocks were selected.

The LSTM network (200 hidden units, tanh activation, sigmoid gate, 0.01 dropout) predicted rest, open, close and reaching movement states every 100 ms. Training used Adam optimizer (0.001 learning rate, 128 batch size, 200 epochs), categorical cross-entropy loss and L2 regularization (0.0001 weight decay). This locked model was used for all subsequent experiments.

### Evaluating locked decoder accuracy

All sessions collected after locking the decoder were included for evaluating the stability of decoding accuracy, consisting of three-class data blocks (*n* = 39) and four-class data blocks (*n* = 6). While the different movement conditions were randomized in case of the three-class blocks, they were in a fixed order of ‘rest > close > reach > open’, mimicking the lifting of an object in case of the four-class blocks. The number of trials per movement condition for the three-class dataset was 15 for hand open, 15 for hand close and either 19 or 31 for rest, depending on the block. The number of trials per condition for the four-class dataset was usually 15 for hand open, 15 for hand close, 15 for movement and 16 for rest. Scatter plot dots represent individual data blocks with box plots summarizing the distribution across blocks.

### Neural activity tuning analysis

To assess tuning of individual electrodes to specific cued movements, we performed a Kruskal–Wallis test. M1 neural activity was recorded while the participant attempted up to 30 trials of each cued movement (rest, open hand, close hand or arm movement). Neural features, that is the integrated broadband power (100–5,000 Hz), was extracted in the temporal window 0–2 s following cue onset and averaged. The Kruskal–Wallis test was used to assess whether the average broadband power in this temporal window differed significantly across task conditions. For electrodes with a significant omnibus test (*α* < 0.05), a Dunnett’s post hoc comparison compared each movement against ‘rest’. An electrode was classified as tuned if at least one movement was observed to be significantly different from ‘rest’.

### Closed-loop fine grasping

Closed-loop grasping using an LSTM decoder (similar as described above but trained only on one session day) was used to predict movement intentions. The decoded output was then wirelessly sent to a custom-built high-resolution neuromuscular electrical stimulator. The stimulator, secured at the wrist, delivered patterned stimulation (0.5-ms long, 10-kHz charge-balanced multiphasic pulses repeated at 50 Hz) through transcutaneous electrode arrays positioned over the muscles in the forearm responsible for finger flexion and extension. To prevent stimulation-based electrical artifacts from affecting the decoder, we created a hardware clock that would trigger the stimulation pulses for all devices and simultaneously send a trigger to the neural signal processor for 11.2 ms, generating a state to halt the data from being used for movement prediction until the amplifiers completely recovered. Tactile force feedback was implemented using Honeywell FMA 5-N force sensors affixed to the thumb and index tips as well as the palm base. These sensors captured the pressure exerted on the hand and transmitted the data to a computer, which used the incoming data as a trigger for sensory stimulation. This approach generated a sensation localized to the corresponding sensor location.

### Closed-loop precision grasping using deep RL

A low-profile, custom AO was developed to provide stronger grasping for object manipulation, while a passive elbow brace, to provide counter resistance, was worn. The nylon body of the AO was designed by 3D scanning (3D Shape) the participant’s hand and forearm, followed by the Spentys (Forest) digital fabrication workflow to ensure anatomical conformity, proper grasp aperture and comfort. Minor alterations were made in Autodesk Fusion to account for the hardware mounted to the device. The AO was 3D printed (Formlabs Fuse 1) using Nylon 12 Powder, selected for its high tensile strength and biocompatibility standards. A 14-kg servo motor (Injora) was mounted on the AO in the middle of the FDS region. The servo was coupled with a magnetic release to a cord that was attached to a 3D-printed custom acrylic ring (Formlabs Form 2) using BioMed Clear Resin. The ring was worn on the middle finger, which was tethered to the index finger, enabling coordinated flexion. The grasp force was measured by a tactile force sensor (Honeywell FMA 5 N) embedded between the AO and back of the thumb interphalangeal joint, such that all grasp forces were applied against the opposing two tethered fingers. Force sensor data were streamed to MATLAB at 50 Hz and were used to drive real-time sensory feedback. On the basis of the S1 mapping, three electrodes were selected for AO sensory feedback, which reliably elicited a strong tactile percept on the palmer side of his index finger.

When grasp forces exceeded an object-specific threshold for 200 ms, ICMS was delivered to these S1 electrodes at 100 µA (pulse width of 300 µs, interphase 100 µs, frequency 100 Hz) for 1–2 s. To avoid stimulation artifacts, neural recording was blanked during stimulation and for an additional 400 ms, during which the AO remained static.

A dual deep Q network (DQN) RL agent was trained offline in a MATLAB Simulink environment. The mechanical properties (stiffness and damping) of the physical AO-hand system were characterized by advancing the AO through multiple eggshell-grasping cycles. These data were curve-fitted to determine the relevant properties. A nonlinear Simulink model was then created and used in a simulation environment to train the DQN RL agent. One part of the reward function was designed such that the RL agent would advance the servo angle. An upper and lower bound for the target force was empirically determined. In simulation, the continuous force, updated at 10 Hz, was compared against these bounds and the RL agent was further rewarded when the force is kept within these bounds. White noise was added to the force to account for variability in the force output from the sensor resulting because of natural grasping behavior. The RL agent learnt how to control the servo angle to hold the force within the specified bounds despite noisy sensor data.

### Press and hold

‘Press and hold’ assessed the blinded participant’s behavioral and neural responses to cued tactile stimulation of the right wrist and fingers (Fig. 5d). The participant’s right hand was secured to a 45° angled board. Stimuli were applied to four randomized target sites with a force transducer (index pad, index volar pad, thumb pad; Fig. 5c) following a 4 s-on, 4 s-off paradigm (12 trials per location). The force transducer consisted of a 10-kg miniature tension/compression DYMH-103 load cell (Shanghai Qiyi) equipped with a thin rubber tip, a Honeywell universal inline amplifier for voltage conversion and a computer running Noraxon MyoMotion. The transducer’s output was fed into an analog EMG channel and displayed on the monitor, allowing the experimenter to apply ~700 g of force. The force sensor was routinely calibrated to maintain accuracy.

The participant reported the location where he felt the sensation, indicating ‘yes’ if he was unsure of the location but felt it and ‘no’ if he did not feel it. Neural data for the press-and-hold task was integrated between 500 and 5,000 Hz to mitigate the effect of speech artifacts in the neural data and were processed as described above.

### S1 CM

For each session day, the most recent Q-tip stroking and sensorimotor tasks were used to select the electrodes for stimulation. The neural data were processed as described above to derive the integrated broadband power, and the MCCs for each electrode were calculated 0–2 s after cue onset for each task, selecting the 16 electrodes with the highest MCC values for each hand/wrist location. Once the press-and-hold experiment started, electrodes selected through imagination (Q-tip stroking videos) were augmented with electrodes that responded to applied pressure.

Stimulation was applied in intermittent stimulation trains followed by rest periods depending on experimental design and session day. The amplitude, pulse width and interphase stimulation parameters were identical to the S1 mapping paradigm. The frequency used for CM was 200 Hz. Stimulation train times varied between 3.69 s and 5.67 s (5th to 95th percentile, maximum of 6 s). The rest duration varied between 1.08 s and 3.79 s (5th to 95th percentile, maximum of 4 s). Stimulation trains and rest periods were interleaved. Other tasks targeted elbow flexion and extension (bicep-and-triceps task) and the opening and closing of the hand (hand open/close). For the bicep-and-triceps task, the same group of electrodes was stimulated each time. For the hand open/close and Q-tip task, cue-driven electrode stimulation depending on the condition was applied. On the basis of the MCC values, 10–16 electrodes (minimum MCC >0.4) were selected for each condition (biceps, triceps, open, close, index tip, thumb tip and wrist), leading to variations in selected electrodes (Extended Data Fig. 7a–c). Performed experiments varied on each session day. Average stimulation time varied between 0 and 25 min per electrode per session day (Extended Data Fig. 7d).

### tSCS with CM

For the tasks where the participant was receiving both tSCS and CM, 10 min of tSCS priming over the C5–C6 spinal root was administered followed by a left elbow flexion-and-extension task (as described above) with tSCS. Next, 5 min of combined tSCS and S1 priming (mirrored stimulation patterns performed in S1 without stimuli video or vibrotactile stimulation) was applied. Following this, a right elbow flexion-and-extension task was performed with continuous tSCS, while cued S1-ICMS was applied only during the active flexion/extension cues (not during rest or interset rest). Both tSCS and S1-ICMS were discontinued after the final right-side set. No cue-driven S1-ICMS was applied during the left-side sets.

### S1 CM with vibrotactile stimulation

When the participant received both CM and vibrotactile stimulation, depending on the cue, electrodes of either the index tip, thumb tip or wrist were stimulated. Simultaneously, vibrotactile stimulation was applied to the same locations and applied using a battery-powered ESP32 microcontroller and four DRV2605L haptic motor controllers (Adafruit Industries), each connected to an Adafruit mini vibrating motor disc (10 mm diameter × 2.7 mm thickness).

### Linear mixed modeling

To explore the effect of CM on the depth of modulation shown by stimulated electrodes when compared with nonstimulated electrodes, we performed linear mixed modeling. For each electrode, the change in modulation depth was computed as the difference in maximum modulation amplitude between the late-day and early-day data blocks (block 2 − block 1). Electrodes were labeled as stimulated or nonstimulated depending on whether they received stimulation between the two blocks. To test whether stimulation increased modulation depth, we fit a linear mixed-effects model

$$y\approx \mathrm{stim}+(1|\mathrm{session})+(1|\mathrm{electrodeID}),$$

where, *y* is the modulation-depth change, ‘stim’ is the fixed effect (0 = nonstimulated electrode, 1 = stimulated electrode), ‘session’ is a random intercept (ID of session day) and ‘electrodeID’ is a random intercept (ID of electrode).

The fixed-effect estimate (*β*) and its 95% confidence interval (CI) provide the population-level stimulation effect.

### Reporting summary

Further information on research design is available in the Nature Portfolio Reporting Summary linked to this article.

## Online content

Any methods, additional references, Nature Portfolio reporting summaries, source data, extended data, supplementary information, acknowledgements, peer review information; details of author contributions and competing interests; and statements of data and code availability are available at 10.1038/s41591-026-04498-0.

## Supplementary information

Supplementary Information Text for Supplementary Videos 1–3 and text and table for Supplementary Table 1.

Reporting Summary

Supplementary Video 1 Participant demonstrates cortically mediated light grasping through NMES.

Supplementary Video 2 Participant demonstrates cortically mediated precision grasping and self-feeding using an AO.

Supplementary Video 3 Participant demonstrates cortically mediated fine grasping with force control using an RL in loop.

## Data Availability

Identifiable patient data are not freely available owing to confidentiality, but de-identified data requests may be directed to the corresponding author. Requests will be reviewed for compliance with confidentiality restrictions, with an estimated response time within 3 weeks.

## Code availability

All custom code (MATLAB 2024b, Python 3.09, SAS 9.4) to produce figures in the paper can be made available on reasonable request to the corresponding author. Requests may be directed to the corresponding author, with an estimated response time within 3 weeks.
