# Supplementary material for: A neuroprosthesis for restoring hand movement and sensation in a person with complete tetraplegia
Source: Nat Med. 2026 Jul 16;32(7):2591–601. doi: 10.1038/s41591-026-04498-0 (PMC13375563; doi:10.1038/s41591-026-04498-0)
Supplement: Supplementary file 1 — Text for Supplementary Videos 1–3 and text and table for Supplementary Table 1. [file 41591_2026_4498_MOESM1_ESM.pdf]

nature medicine

Supplementary information https://doi.org/10.1038/s41591-026-04498-0

A neuroprosthesis for restoring hand movement and sensation in a person with complete tetraplegia

In the format provided by the authors and unedited

Supplementary Information

Bouton_Supplementary_Video_1.mp4

Supplementary Video 1. Participant demonstrates cortically mediated light grasping through neuromuscular electrical stimulation

Bouton_Supplementary_Video_2.mp4

Supplementary Video 2. Participant demonstrates cortically mediated precision grasping and self-feeding using an active orthosis

Bouton_Supplementary_Video_3.mp4

Supplementary Video 3. Participant demonstrates cortically mediated fine grasping with force-control using an RL in loop

Supplementary Table 1. Optimized structural and functional MRI scan parameters for localizing hand regions in the primary motor and somatosensory cortices.

**Structural and functional MRI scan parameters**	
**T1-weighted (T1w) 3D MPRAGE sequence**	
Isotropic resolution	0.8 mm
TR/TE/TI	2400/2.22/1000
Flip angle	8°
In-plane under-sampling (GRAPPA)	2
Acquisition time	7 min
**T2-weighted (T2w) 3D turbo spin echo (SPACE) sequence**	
Isotropic resolution	0.8 mm
TR/TE	3200/564 ms
In-plane under-sampling (GRAPPA)	2
Acquisition time	6 min
**Task fMRI using the CMRR implementation of multiband gradient echo echo-planar imaging (EPI) sequence**	
Isotropic resolution	2.1 mm
TR/TE	751/35 ms
Flip angle	60°
Slices	72
Multiband factor	725
FOV	228 mm × 228 mm
Matrix size	108 × 108
Phase partial Fourier	7/8
Phase encoding direction	anterior-posterior (A-P)
Echo spacing	0.68 ms
Measurements	330 in 4 mins

A pair of reversed polarity (A-P/P-A) spin echo EPI field mapping acquisitions with matched echo train length and echo spacing to the fMRI acquisition were also acquired
