# Supplementary material for: A neuroprosthesis for restoring hand movement and sensation in a person with complete tetraplegia
Source: Nat Med. 2026 Jul 16;32(7):2591–601. doi: 10.1038/s41591-026-04498-0 (PMC13375563; doi:10.1038/s41591-026-04498-0)
Supplement: Supplementary file 2 — Reporting Summary [file 41591_2026_4498_MOESM2_ESM.pdf]

Corresponding author(s): Chad Bouton, PhD

Last updated by author(s): May 27, 2026

Reporting Summary

Nature Portfolio wishes to improve the reproducibility of the work that we publish. This form provides structure for consistency and transparency in reporting. For further information on Nature Portfolio policies, see our Editorial Policies and the Editorial Policy Checklist.

Statistics

For all statistical analyses, confirm that the following items are present in the figure legend, table legend, main text, or Methods section.

n/a	Confirmed	
☐	☒	The exact sample size (*n*) for each experimental group/condition, given as a discrete number and unit of measurement
☐	☒	A statement on whether measurements were taken from distinct samples or whether the same sample was measured repeatedly
☐	☒	The statistical test(s) used AND whether they are one- or two-sided<br>*Only common tests should be described solely by name; describe more complex techniques in the Methods section.*
☐	☒	A description of all covariates tested
☐	☒	A description of any assumptions or corrections, such as tests of normality and adjustment for multiple comparisons
☐	☒	A full description of the statistical parameters including central tendency (e.g. means) or other basic estimates (e.g. regression coefficient) AND variation (e.g. standard deviation) or associated estimates of uncertainty (e.g. confidence intervals)
☐	☒	For null hypothesis testing, the test statistic (e.g. *F*, *t*, *r*) with confidence intervals, effect sizes, degrees of freedom and *P* value noted<br>*Give P values as exact values whenever suitable.*
☐	☒	For Bayesian analysis, information on the choice of priors and Markov chain Monte Carlo settings
☒	☐	For hierarchical and complex designs, identification of the appropriate level for tests and full reporting of outcomes
☒	☐	Estimates of effect sizes (e.g. Cohen's *d*, Pearson's *r*), indicating how they were calculated

Our web collection on statistics for biologists contains articles on many of the points above.

Software and code

Policy information about availability of computer code

Data collection	Neural data was obtained with the NeuroPort System and Central (version 7.7.0) (Blackrock Microsystems, Salt Lake City, UT).Muscle electromyography data was obtained with Noraxon EMG System (Noraxon USA, Scottsdale, AZ).
Data analysis	All data analysis is described in the Methods section. Softwares used: Matlab 2024b, Python 3.09, and SAS 9.4

For manuscripts utilizing custom algorithms or software that are central to the research but not yet described in published literature, software must be made available to editors and reviewers. We strongly encourage code deposition in a community repository (e.g. GitHub). See the Nature Portfolio guidelines for submitting code & software for further information.

Data

Policy information about availability of data

All manuscripts must include a data availability statement. This statement should provide the following information, where applicable:

- Accession codes, unique identifiers, or web links for publicly available datasets
- A description of any restrictions on data availability
- For clinical datasets or third party data, please ensure that the statement adheres to our policy

Data: Identifiable patient data cannot be openly available due to confidentiality, but de-identified data requests may be directed to the corresponding authors (cbouton@northwell.edu). Requests will be reviewed for compliance with confidentiality restrictions with an estimated response time within 3weeks

Code: All custom code (Matlab 2024b, Python 3.09, SAS 9.4) to produce figures in the paper can be made available upon reasonable request to the corresponding author. Requests may be directed to the corresponding authors (cbouton@northwell.edu) with an estimated response time within 3weeks

Research involving human participants, their data, or biological material

Policy information about studies with human participants or human data. See also policy information about sex, gender (identity/presentation), and sexual orientation and race, ethnicity and racism.

Reporting on sex and gender	All data was collected from 1 male participant with a C5 complete spinal cord injury.
Reporting on race, ethnicity, or other socially relevant groupings	No race, ethnicity, or other socially relevant groupings were used.
Population characteristics	The participant is a male in his 40's with a C5 complete spinal cord injury.
Recruitment	Participant candidates were identified through the Northwell Health Physical Medicine & Rehabilitation (PM&R) network. All potential candidates identified by PM&R network were then reviewed by the research team who have diverse backgrounds and areas of expertise to help minimize bias in final selection.
Ethics oversight	The study was conducted under an Investigation Device Exemption (G170200) issued by the Food and Drug Administration. The protocol was approved by the Northwell Health Institutional Review Board (IRB# 17-0840) and was in accordance with the Declaration of Helsinki. The participant provided written informed consent for study participation, and permission for the use of photos, along with audio and video recordings, in which he appears.

Note that full information on the approval of the study protocol must also be provided in the manuscript.

Field-specific reporting

Please select the one below that is the best fit for your research. If you are not sure, read the appropriate sections before making your selection.

☒ Life sciences ☐ Behavioural & social sciences ☐ Ecological, evolutionary & environmental sciences

For a reference copy of the document with all sections, see nature.com/documents/nr-reporting-summary-flat.pdf

Life sciences study design

All studies must disclose on these points even when the disclosure is negative.

Sample size	No statistical methods were used to pre-determine participant sample size since this was a feasibility study. Our sample sizes are similar to those reported in previous publications.<br><br>In all experiments, we determined the initial sample size based on our previous work. As detailed in the manuscript, we used statistical methods to determine if sample size was sufficient to show statistical significance. If needed, based on these analyses, we increased sample sizes and repeated experiments.
Data exclusions	Data was only excluded if they were corrupted or unusable.
Replication	Experimental findings were replicated throughout multiple clinical sessions.
Randomization	Randomization was not used since there was only 1 participant.
Blinding	Blinding was not used due to specific team members often having the expertise to collect and analyze data from a given experiment. However, we always reviewed the analyses and results together as a team to ensure quality and rigor.

Reporting for specific materials, systems and methods

We require information from authors about some types of materials, experimental systems and methods used in many studies. Here, indicate whether each material, system or method listed is relevant to your study. If you are not sure if a list item applies to your research, read the appropriate section before selecting a response.

Materials & experimental systems

n/a	Involved in the study
☒	☐ Antibodies
☒	☐ Eukaryotic cell lines
☒	☐ Palaeontology and archaeology
☒	☐ Animals and other organisms
☐	☒ Clinical data
☒	☐ Dual use research of concern
☒	☐ Plants

Methods

n/a	Involved in the study
☒	☐ ChIP-seq
☒	☐ Flow cytometry
☐	☒ MRI-based neuroimaging

Clinical data

Policy information about clinical studies

All manuscripts should comply with the ICMJE guidelines for publication of clinical research and a completed CONSORT checklist must be included with all submissions.

Clinical trial registration	NCT03680872
Study protocol	See Clinical Trials website: clinicaltrials.gov/study/NCT03680872
Data collection	Data on 1 participant was collected between 2021 to 2025 at the Feinstein Institutes for Medical Research. During this time, the participant attended up to 3 study sessions a week with each session lasting up to 4 hours. Supplementary Table 1 also illustrates the experimental timeline.
Outcomes	Our primary and secondary outcomes were restoration of movement and restoration of sensation, respectively. They were pre-defined using pre-established standards in the clinical community. Sensorimotor tasks were tailored and selected based on their abilities.

Plants

Seed stocks	Not applicable.
Novel plant genotypes	Not applicable.
Authentication	Not applicable.

Magnetic resonance imaging

Experimental design

Design type	Tasked based
Design specifications	Each task consisted of 12 seconds of activity (flexion or stroking), followed by 12 seconds of rest, with 10 repetitions. Additionally, for S1 tasks, a TENS unit (LG TecElite) delivered electrical stimulation (up to 10 mA, 30 Hz, 300 µs pulse width) to the same fingers using ring electrodes (Natus Medical Inc.) placed across the first knuckle. A programmed microcontroller activated solid state relays to target the correct finger and control the stimulation timing (12s ON, 12s OFF) to synchronize with the Q-tip videos. The sensory tasks were performed in 3 varieties: Q-tip video only, TENS only, and both simultaneously
Behavioral performance measures	The participant watched videos and verbalized when the task was completed.

Acquisition

Imaging type(s)	Functional MRI
Field strength	3T
Sequence & imaging parameters	T1w/T2w-based myelin content and cortical thickness maps (see Methods for more details)
Area of acquisition	Sensory and motor cortices

Diffusion MRI ☐ Used ☒ Not used

Preprocessing

Preprocessing software	MRI preprocessing used the HCP minimal preprocessing pipelines (v3.2741) including motion and distortion correction, cortical surface reconstruction and subcortical segmentation, T1w/T2w-based myelin content and cortical thickness maps, fMRI data transformation to MNI and CIFTI grey-ordinate standard spaces using MSMAll-based registration and 6 mm surface smoothing . Spatially specific structured noise was removed using the HCP's multi-run (v4.0) ICA-FIX44–46 with training data for multi fMRI (multiple finger tasks) and linear trends with a high pass filter (>0.1 Hz) without regressing out motion parameters. Somatotopic functional responses were estimated using a generalized linear model (GLM)-based fMRI analysis on the grey-ordinate data space for each finger.
Normalization	See above; see Methods
Normalization template	See above; see Methods
Noise and artifact removal	See above; see Methods
Volume censoring	See above; see Methods

Statistical modeling & inference

Model type and settings	Various methods used; see Methods
Effect(s) tested	See Methods

Specify type of analysis: ☒ Whole brain ☐ ROI-based ☐ Both

Statistic type for inference (See Eklund et al. 2016)	Voxel-wise; see Methods
Correction	Not applicable.

Models & analysis

n/a	Involved in the study
☒	☐ Functional and/or effective connectivity
☒	☐ Graph analysis
☒	☐ Multivariate modeling or predictive analysis
